# A framework for large-scale metabolome drug profiling links coenzyme A metabolism to the toxicity of anti-cancer drug dichloroacetate

**DOI:** 10.1038/s42003-018-0111-x

**Published:** 2018-08-03

**Authors:** Sébastien Dubuis, Karin Ortmayr, Mattia Zampieri

**Affiliations:** Institute of Molecular Systems Biology, ETH Zurich, Auguste-Piccard-Hof 1, CH-8093 Zurich, Switzerland

## Abstract

Metabolic profiling of cell line collections has become an invaluable tool to study disease etiology, drug modes of action and to select personalized treatments. However, large-scale in vitro dynamic metabolic profiling is limited by time-consuming sampling and complex measurement procedures. By adapting a mass spectrometry-based metabolomics workflow for high-throughput profiling of diverse adherent mammalian cells, we establish a framework for the rapid measurement and analysis of drug-induced dynamic changes in intracellular metabolites. This methodology is scalable to large compound libraries and is here applied to study the mechanism underlying the toxic effect of dichloroacetate in ovarian cancer cell lines. System-level analysis of the metabolic responses revealed a key and unexpected role of CoA biosynthesis in dichloroacetate toxicity and the more general importance of CoA homeostasis across diverse human cell lines. The herein-proposed strategy for high-content drug metabolic profiling is complementary to other molecular profiling techniques, opening new scientific and drug-discovery opportunities.

## Abstract (web summary)

Sébastien Dubuis et al. investigated a dynamic metabolic adaptation of five ovarian cancer cells to an anti-cancer drug dichloroacetate. This study finds an unexpected role of coenzyme A metabolism in mediating the toxicity of dichloroacetate, illustrating the power of metabolic drug profiling.

## Introduction

A major bottleneck in drug discovery pipelines is the lack of mechanistic information on the primary targets and downstream secondary effects of selected lead compounds. Large-scale approaches enabling the characterization of cell responses to external perturbations have therefore turned into highly relevant technologies in drug discovery and development^1–4^. Among these approaches, the profiling of drug-induced changes in model organisms at the mRNA and protein level^5,6^ has provided invaluable insights into drug modes of action (MoA)^7–9^, drug–drug interaction mechanisms^10^ and drug repurposing^2,11^. Conceptually similar to transcriptomics and proteomics platforms, metabolomics provides an orthogonal multi-parametric readout aiming at quantifying the full spectrum of small molecules in the cell, the so-called metabolome. Applied to drug discovery research, metabolome profiling of drug-perturbed cell lines in vitro was key in revealing drug modes of action and in identifying potential weaknesses in cellular drug response, as well as genetic polymorphisms associated with drug susceptibility^12–19^.

Metabolomics-based approaches have a notable advantage over existing functional genomics platforms in that they enable an unparalleled throughput^20,21^. However, despite significant advancements in high-resolution mass-spectrometry (MS) profiling of cellular samples^21–23^, efficient experimental and computational workflows for large-scale dynamic metabolome profiling in mammalian cells in vitro are lagging behind. Metabolome screenings that adopt classical metabolomics techniques^24,25^ are often hampered by a limited throughput, laborious sample preparation and the lack of rigorous, yet simple, data analysis pipelines to interpret dynamic metabolome profiles. To address these limitations, our group developed a high-throughput and robust method to perform large-scale metabolic profiling in adherent mammalian cells at steady state^26^, using a 96-well plate cultivation format combined with time-lapse microscopy and flow-injection time-of-flight mass spectrometry^23^ (TOFMS). Here, we extend this methodology to allow rapid sample collection and the analysis of dynamic changes in the intracellular metabolome of diverse mammalian cell lines upon external perturbations. We applied this methodology to profile the diversity of metabolic adaptive responses in five ovarian cancer cell lines to the potential anti-cancer drug dichloroacetate (DCA), and shed light on its mode of action.

The presented framework for in vitro large-scale dynamic metabolomics of perturbed adherent mammalian cell lines is complementary to and scales with high-throughput growth-based phenotypic screens of large compound libraries. Moreover, we provide a proof of principle that our approach can generate testable predictions to elucidate the origin of drug response variability and drug modes of action. Such a platform may complement and improve the translational value of classical in vitro phenotype-based drug screenings^21,27^, and provide insights into the mechanisms of action of small molecules facilitating early stages of drug discovery^28–30^.

## Results

### High-throughput dynamic metabolome profiling of drug action

Large-scale metabolic profiling of transient drug responses among diverse cell types necessitates new methodologies enabling parallelized and rapid sample collection, high-throughput metabolome profiling and an effective normalization approach for metabolomics data. Here, we developed a combined experimental–computational approach enabling the rapid profiling of drug-induced dynamic changes in the baseline metabolic profile of diverse cell lines in parallel. This approach was applied here to study the metabolic responses of five ovarian cancer cell lines to DCA, an activator of pyruvate dehydrogenase (PDH).

**Fig. 1:**
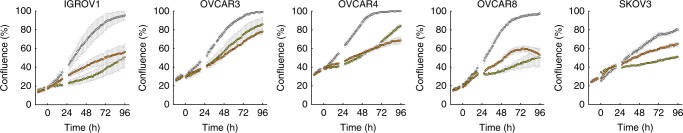
Growth of ovarian cancer cell lines upon drug perturbation. Cell confluence measured by time-lapse microscopy during growth of five ovarian cancer cell lines in RPMI-1640 medium (i.e. untreated condition in gray), and upon dichloroacetate (orange) and oxamate (green) treatments (Supplementary Figure 11). Cell confluence is reported as mean ± SD across three replicates

The five ovarian cell lines IGROV1, OVCAR3, OVCAR4, OVCAR8, and SKOV3 were grown in parallel in 96-well plates for 4 days. Cells were exposed to the corresponding drug dose yielding 50% growth inhibition (GI_50_,Table 1) and metabolomics samples were collected every 24 h following the extraction protocol described in ref. ^26^ and summarized in Supplementary Figure 1. In the present study, nine replicate plates were prepared: one plate served to continuously monitor cell growth via cell confluence by time-lapse microscopy using an automated multi-well plate reader (Fig. 1), while the remaining plates were used for metabolome extraction immediately before, and at 24, 48, 72 and 96 h after drug exposure (Supplementary Figure 1). At each sampling time point, one plate was used to generate cell extract samples, while the second plate served to determine extracted cell numbers per well using bright-field microscopy^26^. Cell extract samples were profiled by flow injection analysis (FIA) and TOFMS (FIA–TOFMS) as described previously^23^, enabling high-throughput analysis of large sample collections. The detected ions were annotated based purely on the accurate mass, and by assuming that deprotonation is the most frequent and reliable form of ionization in negative mode. By matching measured *m*/*z* against calculated monoisotopic masses of metabolites listed in the Human Metabolome Database (HMDB^31^) and in the genome-scale reconstruction of human metabolism (Recon 2^32^), we putatively annotated 2482 ions (Supplementary Data 1). Importantly, in absence of prior chromatographic separation, FIA-TOFMS cannot distinguish isobaric metabolites, as well as in-source fragments that are detected at the identical exact mass.

**Table 1:** Drug concentrations used in metabolomics experiments

Cell line	Oxamate dose (mM)	Dichloroacetate dose (mM)
IGROV1	13	11
OVCAR3	40	12
OVCAR4	18	25
OVCAR8	6.7	25
SKOV3	31	25

The given concentrations correspond to the effective concentration in the medium

To estimate time-dependent (e.g. drug-induced) changes in intracellular metabolite abundances from non-targeted metabolomics data, we here developed a regression-based analysis approach to compare transient changes in the metabolome of drug-treated cells against steady-state unperturbed cell metabolic profiles (Supplementary Figure 2) determined following the approach described in ref. ^26^ and here briefly summarized.

By definition, the intracellular concentrations of metabolites at steady state are constant in time. Hence, in samples from unperturbed growing cells for each metabolite *i* in cell line *j*, measured intensities, *I*_*j*,*i*_, scale proportionally with the metabolite abundance in the cell [*m*_*i*_], times the extracted cell number (*N*_c_, derived from bright-field microscopy^26^, see Supplementary Figure 3):

1 $$I_{j,i} \propto N_{\mathrm{c}} \cdot \left[ m_i \right]_j$$

Hence, we can model the measured metabolite intensities in a given cell line as follows:

2 $$I_{j,i} = \alpha _{j,i} \cdot N_{\mathrm{c}} + \beta _i,$$

where *β*_*i*_ is an offset value corresponding to the experimental MS background signal, and *α*_*j*,*i*_ represents the abundance of metabolite *i* per cell. Notably, *α*_*j*,*i*_ contains an unknown scaling factor that is reflective of the fundamental proportionality between metabolite concentration and MS signal intensity. For each metabolite, we use a multiple regression scheme to fit the linear model and regress the cell line-specific *α* values and the offset *β* across all cell lines at once (Supplementary Figure 4). To assess the reliability of the parameter estimates, each fitted parameter is associated with a *p*-value (*F* statistic with the hypothesis that the coefficient is equal to zero). It is worth noting that this procedure allows systematically filtering out annotated ions which are unlikely to originate from extracted cells because the measured ion intensity does not exhibit any dependency with the cell number, as well as ions for which the measured intensities are below the detection limit, and the estimated cell line-specific *α* values are below or close to 0. Out of the 2482 ions annotated in the ovarian cancer cell dataset, we obtained relevant parameter estimates for 1546 putatively annotated ions, i.e. *α* > 0 and *p* ≤ 0.001 in at least one cell line, with a median coefficient of variation of 17.4% (Supplementary Figure 5).

**Fig. 2:**
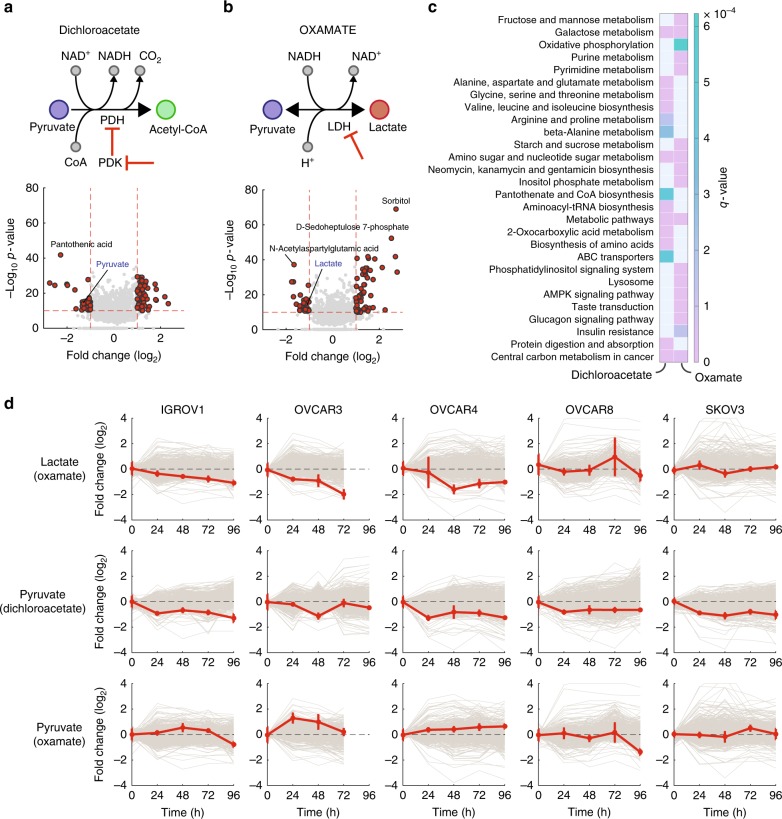
Analysis of transient dynamic metabolic changes upon external perturbation. **a** and **b** Schematic representation of the enzymatic reactions targeted by oxamate and dichloroacetate, and volcano plots summarizing overall metabolome changes. Each dot corresponds to a metabolite. The negative log_10_ of the product between minimum *p*-values over the time course across the five cell lines is plotted against the median of maximum fold-changes. Metabolites highlighted in red have an absolute log_2_ fold-change >1 and a *p*-value ≤ 1e−10. **c** KEGG pathway enrichment of metabolites consistently affected by dichloroacetate and oxamate treatments, highlighted in panel **b**. Only enriched pathways with a significance corrected for multiple tests *q*-value (Storey) ≤ 0.001 are considered. **d** Time-dependent fold changes (red line) of lactate upon oxamate treatment, and pyruvate upon oxamate and dichloroacetate treatments. Data are the mean ± SD of three replicates. The profiles of all other detected metabolites are shown in gray

To evaluate dynamic metabolite changes upon an external perturbation, we calculate time-dependent fold-change values for each metabolite based on the parametrized model derived from steady-state unperturbed metabolome profiles. For each metabolite *i* and time point *t* after exposure to treatment *D*, we estimate the deviation of metabolite abundance from the unperturbed steady-state condition as follows:

3 $${\mathrm {FC}}_{t,i}^{D,j} = {\mathrm{log}}_2\left( {\frac{{I_{t,i}^{D,j}}}{{\alpha _{j,i} \cdot N_{j,t} + \beta _i}}} \right)$$

where $I_{t,i}^{D,j}$ is the intensity measured for metabolite *i* at time *t* after exposure of cell line *j* to compound *D*. $I_{t,i}^{D,j}$ is compared to the corresponding theoretical steady-state unperturbed metabolite intensity (denominator in Eq. (3)) which is calculated from the previously estimated *α* and *β* parameters and the extracted cell number at the time of sampling, *N*_*j*,*t*_, derived from bright-field microscopy images (Supplementary Figures 1 and 3). For each time point, the difference between measured and expected metabolite intensities is expressed in log_2_ fold-changes, and significance is quantified by means of *p*-values from *t*-test analysis. In the following, our metabolome profiling pipeline was applied to investigate the metabolic response to the small-molecule agent DCA (Fig. 2a and b), in the five ovarian cancer cell lines IGROV1, OVCAR3, OVCAR4, OVCAR8, and SKOV3.

### Interplay of DCA MoA and coenzyme A (CoA) metabolism

DCA is a mitochondria-targeting small molecule that activates PDH by inhibiting pyruvate dehydrogenase kinase (PDK)^33^. By blocking PDH phosphorylation, DCA favors increased flux of pyruvate into mitochondria^34^. To date, the exact mechanism by which DCA is toxic to cancer cells has remained unclear^35^. Recent studies suggested that the activation of PDH diverts metabolism from fermentative glycolysis to oxidative phosphorylation, leading to a loss in mitochondrial membrane potential and a reopening of voltage-sensitive and redox-sensitive mitochondrial transition pores, which ultimately triggers an apoptotic cascade in cancer cells^33^.

**Fig. 3:**
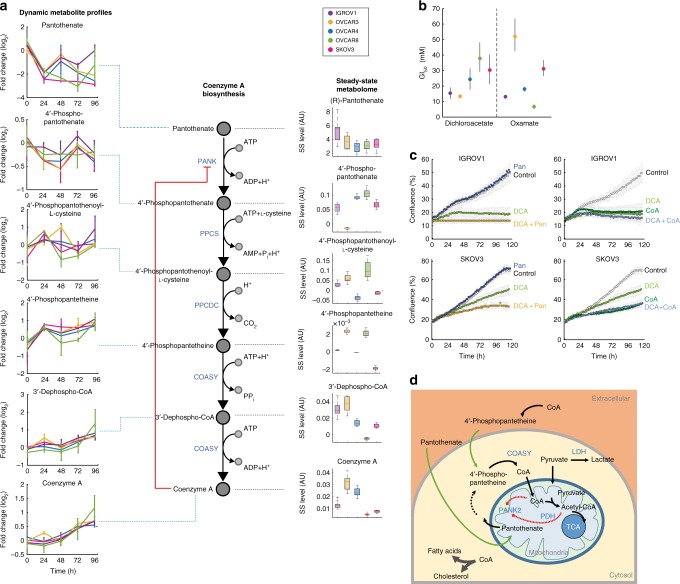
Influence of CoA metabolism on dichloroacetate action. **a** Schematic representation of the CoA biosynthetic pathway. Pantothenate kinases (PANK) catalyze the rate-limiting step in CoA biosynthesis^39^. For each pathway intermediate, relative steady-state (SS) abundances in the untreated condition (right-hand side) and dynamic changes upon dichloroacetate treatment (left-hand side) are shown. Steady-state levels of pathway intermediates are represented as the distribution of residuals in the linear fitting of raw MS measurements multiplied by the inferred cell line specific *α* values. **b** Determined GI_50_ concentrations for oxamate and dichloroacetate across cell lines (Supplementary Figure 11). **c** IGROV1 and SKOV3 cells were grown in RPMI-1640 medium before addition of perturbing agents and continuous confluence monitoring for ∼5 days. Six conditions were tested: normal RPMI-1640 medium (Control), addition of 2.1 µM pantothenate, with and without 11 mM (IGROV1) or 25 mM (SKOV3) of dichloroacetate (Pan/Pan + DCA), addition of 100 µM CoA with and without 13 mM (IGROV1) or 31 mM (SKOV3) of dichloroacetate (CoA/CoA + DCA). **d** Schematic representation of CoA metabolism. CoA plays a central role in energy and fatty acid metabolism, acting as an acyl group carrier to form acetyl-CoA and other important compounds, such as fatty acids, cholesterol, and acetylcholine. PANK2, the first and rate-limiting metabolic enzyme in the CoA biosynthetic pathway, is allosterically regulated^38,42^ and localizes in the mitochondrial inter-membrane space^41,42^. CoA is produced in the cytosol and subsequently actively transported into the mitochondrial matrix. Alternatively, can access CoA from the extracellular environment thanks to the action of extracellular ectonucleotide pyrophosphatases contained in the serum^70^. These enzymes cleave the CoA molecule to form 4’-phosphopantetheine, which can enter the cells one enzymatic step above CoA formation by COASY

Despite large differences in doubling times (Supplementary Figure 6), degrees of invasiveness^36^ and metabolic phenotypes^37^ (Supplementary Figure 7), the five selected ovarian cancer lines exhibited common metabolic adaptive changes to DCA exposure. In our dynamic metabolome data, we observed a consistent reduction in pyruvate levels across all cell lines upon DCA treatment (Fig. 2d), and a reduction in lactate secretion (Supplementary Figure 8). Strikingly, the most significant metabolic change (*p*-value 5.7e−43) across all five cell lines was a marked depletion of intracellular pantothenate (Figs. 2a and 3a), which was additionally confirmed by quantitative LC–MS/MS measurements (Supplementary Figure 9). While pyruvate depletion and reduced lactate secretion are likely direct consequences of PDH activation, a depletion of pantothenate and a concomitant increase in the total pools of CoA (Fig. 3a and Supplementary Figure 10) hints at an unexpected activation of de novo CoA biosynthesis.

Pantothenate is the primary precursor required for CoA biosynthesis. CoA in turn regulates its own biosynthesis via allosteric inhibition of the first enzymatic step in the pathway, catalyzed by mitochondrial pantothenate kinase 2 (PANK2)^38,39^ (Fig. 3a, d). While human PANK2 locates in the inner mitochondrial membrane^40,41^, the remaining CoA biosynthetic steps take place in the cytoplasm. Notably, CoA pools in the different cellular compartments are tightly regulated, such that typical CoA concentrations are 1–2 orders of magnitude higher in mitochondria (∼2–5 mM) than in the mitochondrial intermembrane space and the cytosol (∼0.02–0.14 mM)^42,43^. We hypothesize that a hyper-activation of PDH in the mitochondrial matrix could entail a depletion of CoA in the immediate surroundings of PANK2, hence lifting allosteric inhibition of de novo CoA biosynthesis. In such a scenario, our observations are consistent with an attempt of DCA-treated cells to re-equilibrate CoA levels across compartments by increasing CoA biosynthesis (Fig. 3a). According to this model, the resulting increased pantothenate phosphorylation would explain the observed depletion of intracellular pantothenate, and the parallel accumulation of total CoA in the cells^44^ (Fig. 3a). Moreover, we observed the largest amount of intracellular pantothenate at steady state (Fig. 3a) in the two cell lines with the highest sensitivity (i.e. lowest GI_50_) to DCA, OVCAR3 and IGROV1 (Fig. 3b and Supplementary Figures 11 and 16), supporting a functional association of CoA metabolism with the MoA of DCA.

To test our hypothesis, we selected IGROV1 and SKOV3 cells, which exhibit different steady-state levels of pantothenate and a distinctly different sensitivity to DCA (Fig. 3b). We monitored the growth of IGROV1 and SKOV3 cells upon DCA treatment, with and without supplementing the medium with 2.1 µM pantothenate or 100 µM CoA (Fig. 3c). We found that increasing extracellular pantothenate concentration strongly aggravated the toxicity of DCA in both cell lines (Fig. 3c), while being neutral for cells in normal RPMI-1640 medium (containing 0.25 µM pantothenate). Surprisingly, even in the absence of DCA, supplementing CoA to the medium had a strong toxic effect on cells. Cells supplemented with 100 or 500 µM CoA (Fig. 3c and Supplementary Figure 12) exhibited a first phase of normal growth, followed by rapid growth arrest (Fig. 3c). Interestingly, supplementation of CoA completely masked DCA toxicity when co-administered (Fig. 3c and Supplementary Figure 12).

The synergistic effect of pantothenate with DCA, and the antagonistic interaction of DCA with CoA reinforce our premise of a functional interplay between CoA metabolism and cell growth inhibition caused by DCA (Fig. 3d). Because CoA biosynthesis is regulated (i.e. repressed) immediately downstream of pantothenate (Fig. 3a), CoA levels can be controlled in spite of high pantothenate concentrations. Hence, supplementing pantothenate to the medium has no toxic effect to cells (red curve in Fig. 3c). However, when cells are additionally challenged with DCA, CoA biosynthesis is activated and higher levels of pantothenate can lead to higher CoA biosynthetic flux (orange curve in Fig. 3c). To verify our conclusions, we monitored metabolome changes in IGROV1 cells upon addition of 2.1 µM pantothenate or 200 µM CoA. Consistent with our expectations, supplemented pantothenate is internalized but metabolism is otherwise unperturbed, while CoA addition induces pleiotropic changes in intracellular metabolite abundances, indicating a large deviation from metabolic steady state (Supplementary Figure 13). We concluded that by directly providing CoA extracellularly we bypassed the main control mechanism for CoA homeostasis and in turn impaired cell growth.

### Model-based analysis of DCA MoA and CoA toxicity

**Fig. 4:**
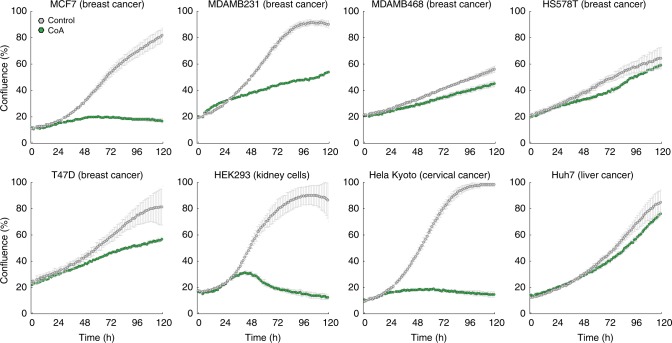
CoA toxicity across multiple cell lines. Cell lines from breast, kidney, cervical and liver tissues were grown with (green) and without (gray) the addition of 100 µM CoA. Cell confluence is reported as mean ± SD across three replicates

**Fig. 5:**
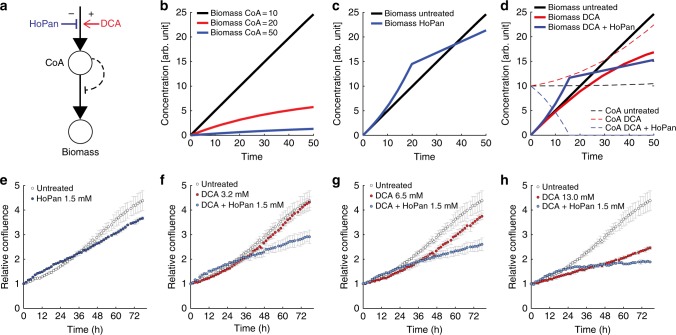
Mechanistic model of CoA-mediated toxicity of dichloroacetate. **a** Schematic overview of the minimal model, where CoA production can be increased by dichloroacetate (DCA), or reduced by hopantenate (HoPan). CoA is directly used to form new biomass, which in turn CoA can inhibit by an undefined mechanism. **b** CoA inhibits growth in a concentration-dependent manner, while (**c**) HoPan inhibition of the first steps in CoA biosynthesis can reduce CoA levels and have an initial beneficial effect on growth, until CoA biosynthesis becomes growth-limiting. **d** On the contrary, DCA inhibits growth (bold red line) by virtue of increasing CoA levels (dashed red line). Addition of HoPan initially restores growth (bold blue line) by reducing CoA levels (dashed blue line). **e**–**h** Experimental verification of the model. IGROV1 cells were supplemented with HoPan (**e**), or increasing concentrations of DCA, in presence or absence of 1.5 mM HoPan (**f**–**h**). Growth is monitored continuously using cell confluence measurements. Relative confluence data reported are mean ± standard deviation across three replicate wells, normalized to the initial confluence, while the kinetic parameters used for model simulations can be found in the Methods section

We next asked whether the observed growth inhibitory effect of CoA was restricted only to ovarian cancer cell lines. To this end, we tested the effect of CoA on seven additional cancer cell lines from different tissue types, and one non-cancer cell line (HEK293 kidney cells). Despite distinct differences in sensitivity, all cell lines exhibited growth reduction upon supplementation of culture media with 100 µM CoA (Fig. 4). To better understand the interplay between biosynthesis/utilization of CoA and DCA toxicity, we created a minimal kinetic model that consists of only two reactions following Michaelis–Menten kinetics: biosynthesis of CoA (Eq. (4)) and CoA utilization for biomass production (Eq. (5), Fig. 5a), assuming a growth inhibitory activity of CoA:

4 $$v_{{\mathrm{CoA}}} = v_{{\mathrm{CoA}}_{{\mathrm{max}}}} \cdot \left( {\frac{{\left[ {\mathrm {{DCA}}} \right]}}{{\left[ {\mathrm {{DCA}}} \right] + K_{{\mathrm{DCA}}}}} + 1} \right)$$

5 $$v_{{\mathrm{biomass}}} = v_{{\mathrm{biomass}}_{{\mathrm{max}}}} \cdot \left( {\frac{{\left[ {\mathrm {{CoA}}} \right]}}{{\left[ {\mathrm {{CoA}}} \right] + K_{{\mathrm{CoA}}} \cdot \left( {1 + \frac{{\left[ {\mathrm {{CoA}}} \right]^3}}{{K_{\mathrm {i}}}}} \right)}}} \right)$$

where *v*_CoA,max_ and *v*_biomass,max_ represent the corresponding maximum flux capacities, [DCA] and [CoA] are the concentrations of DCA and CoA, respectively, *K*_DCA_ and *K*_CoA_ are the Michaelis–Menten constants, and *K*_i_ is the inhibitory constant for CoA. The model assumes that in unperturbed cells, CoA is not limiting for biomass production (i.e. [CoA] ≫ *K*_m_), and that cells are ultrasensitive to high levels of CoA, which in turn inhibit biomass production (i.e. *K*_I_ ≫ *K*_m_).

This simple model is able to qualitatively recapitulate the toxic effect of increasing CoA levels either as a consequence of increased CoA biosynthesis upon DCA treatment or external addition of CoA (Fig. 5b). In addition, the model can be used to predict the effect of a reduced CoA biosynthetic flux (e.g. reduced *v*_CoA,max_). In such a scenario, the model qualitatively predicts two phases: a first one where reduction of intracellular CoA levels increases biomass production, and a second phase in which CoA biosynthesis becomes limiting for growth (Fig. 5c). A qualitatively similar behavior is expected when CoA biosynthesis is inhibited in the presence of DCA (Fig. 5d).

To test the model predictions, we supplemented IGROV1 cells with different concentrations of DCA, in the presence or absence of 1.5 mM hopantenate, an inhibitor of the first enzymatic step in CoA biosynthesis. In agreement with our minimal model, cells with hopantenate exhibit an initially higher proliferation rate with respect to unperturbed cells, before entering in a second phase of reduced growth (Fig. 5e–h). Moreover, in the first phase hopantenate fully reverted the growth inhibitory effect of DCA, allowing cells to grow at similar if not higher rates as untreated cells, hence confirming that enhanced CoA biosynthesis is at the core of the mode of action of DCA. Overall, these experimental results are consistent with our minimal model, and emphasize the importance of CoA homeostasis and its role in mediating DCA toxicity. To our knowledge, this is the first time that a toxic effect of CoA in mammalian cells has been shown and was linked to the mode of action of DCA.

Consistent with our in vitro results, in vivo inhibition of PANK in mice by hopantenate resulted in 167-fold higher expression of PDK^45^. This observation suggests dichotomous compensatory mechanisms to regulate CoA homeostasis: inhibition of CoA biosynthesis activates PDK, which in turn represses PDH^45^, while inhibition of PDK by DCA has the opposite effect, and promotes CoA biosynthesis. The resulting over-induction of cytosolic de novo CoA biosynthesis can in turn aggravate DCA toxicity. It is important to note that the mechanism by which enhanced CoA biosynthesis inhibits growth remains unclear. It is possible that rather than CoA directly, it is the accumulation of an intermediary product of CoA metabolism or the hyper-utilization of CoA^46,47^ that becomes toxic to cells. To test whether the observed adaptive response to DCA was an indirect effect associated with a general stress response upon growth inhibition and/or reduced lactate secretion, we tested the effect of oxamate, a small molecule that inhibits the conversion of pyruvate into lactate.

### Oxamate elicits different metabolic responses

Oxamate is a competitive inhibitor of lactate dehydrogenase (LDHA) with respect to pyruvate^48^ (Fig. 2b). A growing body of evidence indicates that oxamate induces apoptosis exclusively in cancer cells^48,49^. According to current theory, the inhibition of lactate production, together with typically high glycolytic rates in cancer cells, causes an over-production of toxic superoxide by the mitochondrial electron transport chain. Since both drugs decrease lactate secretion rates (Supplementary Figure 8), oxamate treatment could lead to similar metabolic adaptive mechanisms as DCA.

In our dynamic metabolome profiling data, we observed a significant accumulation of intermediates in TCA cycle upon oxamate treatment, and a concomitant reduction of intracellular ATP levels, in accordance with previous findings^50^. In particular, we observed a consistent and large accumulation of sorbitol and sedoheptulose 7-phosphate (Fig. 2b). Overall, we found that the significant metabolic changes common to all cell lines locate in central metabolic pathways like oxidative phosphorylation and nucleotide metabolism (Fig. 2c). Taken together, changes induced by oxamate were largely different from those induced by DCA (Fig. 3a–c, Supplementary Figure 14), suggesting for radically different metabolic adaptive strategies. Unlike PDH activation by DCA, inhibition of LDHA seems to redirect intermediates in upper glycolysis to other pathways, such as NADPH-dependent reduction of glucose to form sorbitol, or the pentose phosphate pathway (as indicated by accumulation of sedoheptulose 7-phosphate, Fig. 2b). Both metabolic responses are known to counteract oxidative stress^51,52^. Interestingly, we also observed a marked reduction in the levels of N-acetylaspartic acid (Fig. 2b), a potent oxidative stress agent^53^ associated with poor prognosis in ovarian cancer^54^. The marked differences between metabolic adaptive responses to oxamate and to DCA reinforce our previous observation of a selective functional link between CoA metabolism and the mode of action of DCA.

## Discussion

In this study, we present a novel experimental and computational workflow for high-content dynamic metabolome profiling that enables a systematic and high-throughput investigation of dynamic changes in the intracellular metabolism of adherent mammalian cells upon environmental perturbation. Our methodology provides a novel way to perform high-throughput dynamic metabolic screens in adherent cell lines, facilitated by a miniaturized parallel 96-well cultivation system, a simple and rapid metabolite extraction procedure and automated time-lapse microscopy^26^. We additionally exploit the unique throughput advantages of flow-injection high-resolution MS-based metabolomics which has become an invaluable tool^55–58^ for exploratory studies and the profiling of large sample collections. Altogether, our methodology offers new scientific and clinical opportunities for large-scale in vitro exploratory metabolome drug screenings and a complementary tool to more targeted approaches^59^. Of note, as compared to more conventional LC–MS-based approaches, even moderately sized studies can benefit from our exploratory methodology, given the extended metabolic and chemical space covered, the reduced complexity of sample preparation, the rapid measurement and the automatized acquisition of normalization parameters.

A major challenge common to many high-content screenings, and particularly relevant for non-targeted approaches, is the computational analysis of large datasets for the generation of testable predictions. Here, we implemented a systematic data processing and analysis pipeline that allows comprehensively interpreting dynamic metabolic profiles and extracting the most informative features (Supplementary Figure 2). While changes in metabolite abundance do not necessarily correspond to changes in conversion rates (i.e. fluxes), altered metabolite pools can be reflective of functional changes in the cell^60^. By investigating the dynamic responses to the perturbing agents DCA and oxamate, we proposed a previously undescribed role of CoA metabolism in mediating the toxicity of DCA. Therapeutically, high dosages of DCA are needed in order to effectively suppress tumor growth^35^, limiting further development and usage of this compound in clinics. Nevertheless, our results suggest that compounds affecting CoA production are likely to exhibit strong epistatic interactions with DCA. In light of the promising initial evidence that we provided here, this possibility warrants more attention in future studies.

We have shown here that our experimental and computational framework for high-throughput drug metabolome profiling can provide key insights into the cellular response to bioactive compounds. As such, this technique can become a powerful complementary tool to aid lead selection at early stages of drug discovery, and to predict compound modes of action^61^, similar to approaches exploiting large compendia of cellular gene expression profiles^19^. For instance, comparative analysis can reveal uncharacterized compounds featuring metabolic responses similar to drugs with known molecular targets^2^. Our proof-of-principle example illustrates how in vitro high-content metabolic drug profiling can provide a first coarse-grained characterization of a compound mode of action and guide the design of follow-up experiments in clinically relevant models, aiming at a mechanistic understanding of drug action. Despite the difficulty in translating the relevance of in vitro phenotypes into in vivo outcomes^62^, we envisage that this approach can be applied to the profiling of large sets of bioactive compounds^28^ in a large cohort of cell lines^63,64^. In such a setting, this methodology can potentially deliver invaluable insights to highlight mechanistic biomarkers to be tested in vivo, to resolve the functionality of genetic variations, and to understand the interplay between the drug mode of action and intrinsic cell-to-cell tolerance variability.

## Methods

### Cell cultivation

The ovarian cancer cell lines IGROV1, OVCAR3, OVCAR4, OVCAR8, and SKOV3 were obtained from the National Cancer Institute (NCI, Bethesda, MD, USA) and maintained according to standard protocols at 37 °C with 5% CO_2_ in RPMI-1640 (Biological Industries, cat. no. 01-101-1A) supplemented with 2 mM l-glutamine (Gibco, cat. no. 25030024), 2 g/L d-glucose (Sigma Aldrich, cat. no. G8644), 100 U/mL penicillin/streptomycin (Gibco, cat. no. 15140122), and 5% fetal bovine serum (FBS, Sigma Aldrich, cat. no. F6178). After thawing, the cells were expanded in standard cell culture flasks (Nunc T75, Thermo Scientific). After one week, the cells were transferred to fresh medium where FBS was replaced by dialyzed FBS (Sigma Aldrich, cat. no. F0392) in order to facilitate metabolite quantification. Cells were maintained in this medium with dialyzed FBS for the remaining duration of the experiment. Three of the cell lines (IGROV1, OVCAR3, OVCAR4) were exemplarily tested for mycoplasma contamination, and were confirmed mycoplasma-free. Overall, the cell lines were expanded in T75 for a total of 3 weeks from thawing until perturbation and metabolomics experiments. With the aim of determining the starting cell density for the experiment, a preliminary cultivation in 96-well cell culture plates was done one week prior to the experiment. To this end, for each cell line, 150 µL of eight different dilutions containing different starting cell numbers were plated in triplicate in a 96-well plate. After 72 h, all wells were imaged using a Spark™ 10M (TECAN) and confluence was determined in each well. The optimal starting cell density was subsequently calculated so as to obtain 80% confluence after 72 h.

### Cell growth and segmentation

All procedures for cell growth monitoring and image analysis were adopted from ref. ^29^, and are here briefly summarized. A TECAN Spark 10M plate reader was used to monitor live adherent cell cultures directly in the 96-well culture plate. The choice of image acquisition frequency depends on how fast are the expected growth dynamic changes. Here, we selected a time frequency of 1.5 h as a reasonable tradeoff between the fastest doubling time among our cell lines (∼20 h) and the time it takes to acquire the images for a full plate on the TECAN plate reader (∼30 min). It is worth noting that our procedure can be adapted to other commercially available plate readers. Full detail on bright-field image processing and the extraction of cell confluence and average adherent cell size is described in ref.^26^ (MATLAB code available for download), and is summarized in Supplementary Figure 3.

### Perturbation experiments

Sodium oxamate and sodium DCA were obtained from Sigma Aldrich (cat. no. O2751 and 347795, respectively), and stock solutions of 400 mM oxamate and 250 mM DCA were prepared in distilled water. To determine the GI_50_ drug concentrations, nine different concentrations of oxamate and DCA were tested (Supplementary Figure 10). For each cell line, cells were seeded in 135 µL of fresh medium in 96-well plates according to the previously calculated optimal density. After 24 h, oxamate and DCA, dissolved at different concentrations in 15 µL of medium, were added to the cells in triplicates. Immediately upon drug addition, 24, 48, and 72 h after drug exposure, all wells were imaged using a Spark™ 10M (TECAN) plate reader, and the cell confluence was determined. For each condition and cell line, the growth rate was obtained by fitting an exponential curve to the cell confluence measurements. After calculating the growth rate reduction relative to the untreated condition for each drug concentration, and fitting a sigmoidal curve to the degree of growth inhibition across drug concentrations (shown in Supplementary Figure 10), the GI_50_ was estimated from the fitted curve as the drug concentration causing a 50% reduction in growth rate (Fig. 3b).

### Metabolomics experiments

Cell lines were plated in nine 96-well plates according to the optimal density previously calculated, using 135 µL of medium. To minimize the effect of evaporation, the outmost rows and columns of the plate were omitted, and filled with PBS instead. After 24 h, cells were perturbed with 15 µL of medium containing drug concentrations close to the respective GI_50_ for each cell line. When the calculated GI_50_ dose could not be reached due to limited solubility, the highest concentration possible was used (400 and 250 mM for oxamate and DCA, respectively). 15 µL of fresh medium without drug addition were used as a control. The final concentrations used for each drug and each cell line are given Table 1.

### Sample collection and metabolite extraction

The metabolomics sampling procedure was adapted from an experimental workflow for steady-state metabolome profiling described in ref. ^26^, and is here briefly summarized. Samples were collected immediately before, and at 24, 48, 72, and 96 h after drug addition. Two replicate 96-well plates were processed at each sampling time point (plate A and plate B, see also Supplementary Figure 1). In plate A, the cell culture medium was aspirated from all wells using a multichannel aspirator, and 150 µL of ammonium carbonate (75 mM, pH 7.4, 37 °C) was gently added to each well using a multichannel dispensing pipet. Immediately after aspiration of the washing solution, 100 µL of cold extraction solvent (40% methanol, 40% acetonitrile, 20% water, 25 µM phenyl hydrazine^65^, −20 °C) were added to each well using a multichannel pipet. Plates were sealed with aluminum adhesive to prevent evaporation, immediately transferred to −20 °C for 1 h, and subsequently stored at −80 °C until further processing. In plate B, the cell culture medium was aspirated, and the cells were washed with ammonium carbonate (75 mM, pH 7.4, 37 °C). After aspiration of the washing solvent, 150 µL of PBS (Gibco, cat. no. 10010015) were added to each well, and cell confluence was immediately measured in all wells using a Spark™ 10M (TECAN) plate reader, adopting bright-field microscopy. The cell confluence from plate B was later used to derive extracted cell numbers for normalization, using previously determined average adherent cell sizes (Supplementary Figure 3). Before injection in the mass spectrometer, the 96-well plates were briefly thawed on ice, and the bottom of all wells was scratched using a multichannel pipet with wide-bore tips in order to disrupt and detach all cells from the well bottom. The plates were centrifuged (4 °C, 4000 rcf), and the supernatant was transferred to fresh 96-well plates for FIA-TOFMS measurements.

### FIA-TOFMS analysis

FIA-TOFMS analysis was performed as described in ref. ^23^ on an Agilent 6550 iFunnel Q-TOF LC/MS System (Agilent Technologies, Santa Clara, CA, USA) equipped with an electrospray ion source operated in negative ionization mode. In this setup, the samples are injected into a constant flow of an isopropanol/water mixture (60:40, v/v) buffered with 5 mM ammonium carbonate at pH 9 using a Gerstel MPS2 autosampler (5 µL injection volume). Two compounds were added to the solvent for on-line mass axis correction: 3-amino-1-propanesulfonic acid, (HOT, 138.0230374*m*/*z*, Sigma Aldrich, cat. no. A76109) and hexakis(1H,1H,3H-tetrafluoropropoxy)phosphazine (940.0003763*m*/*z*, HP-0921, Agilent Technologies, Santa Clara, CA, USA). The ion source parameters were set as follows: 325 °C source temperature, 5 L/min drying gas, 30 psig nebulizer pressure, 175 V fragmentor voltage, 65 V skimmer voltage, 750 V octopole voltage. The TOF detector was operated in 4 GHz high-resolution mode with a spectral acquisition rate of 1.4 spectra per second. Mass spectra were recorded in the mass range 50–1000*m*/*z*. Alignment of MS profiles and picking of centroid ion masses were performed using an in-house data processing environment in Matlab R2015b (The Mathworks, Natick)^23^.

### Ion annotation

The ion annotation process is based on a list of known metabolites, compiled from the HMDB^34^ and the Recon2 genome-scale reconstruction of human metabolism^32^. In order to allow annotation of α-keto acid derivatives formed in presence of phenyl hydrazine^65^ in the extraction solvent, we added the sum formulae for the phenylhydrazones (+C_6_H_8_N_2_ −H_2_O) of a total of 30 α-keto acid compounds (selected via KEGG SimComp search http://www.genome.jp/tools/simcomp/) to the metabolite list for annotation. The monoisotopic mass is calculated for each of the listed metabolites based on its sum formula. A list of expected ion masses corresponding to the listed metabolites is subsequently generated, considering only ionization by deprotonation (−H+) in negative mode electrospray ionization. Subsequently, these theoretical ion masses are searched against the detected ion mass-to-charge ratios (*m*/*z*) within a tolerance of 0.003 amu. The final list of annotated ions is compiled considering the best metabolite match (i.e. smallest difference to the expected mass) for each ion.

### Data processing and computational analysis for steady-state metabolome data

All steps of data processing and further analysis were performed in Matlab 2015b (The Mathworks, Natick). For steady-state metabolome profiles, the bioinformatics pipeline is described in ref. ^26^ and is here summarized. Multiple regression analysis to estimate the relative metabolite concentrations at steady state was performed using the Matlab fitlm function. This function infers model parameters *α* (cell line-specific) and *β* by minimizing the Euclidian distance between measured metabolite intensities and model predicted ones. It is worth noting that the *β* represents the MS background signal, or in other words the ion intensity when no cells are extracted. Hence, this particular parameter is independent from cell types. Because of the difficulties in reliably estimating the extracted cell number from bright-field microscopy images above a confluence of 80% (Supplementary Figure 3), and the observed deviation from metabolic steady state (Supplementary Figure 4), we excluded all metabolome measurements taken above this cell density threshold. For each metabolite, we solve the following linear model:

6 $$\left[ {\begin{array}{*{20}{c}} {I_{{\mathrm{cell}}_1,1}} \\ {I_{{\mathrm{cell}}_1,2}} \\ {I_{{\mathrm{cell}}_1,3}} \\ \ldots \\ \ldots \\ {I_{{\mathrm{cell}}_2,1}} \\ {I_{{\mathrm{cell}}_2,2}} \\ {I_{{\mathrm{cell}}_2,3}} \\ \ldots \\ {I_{{\mathrm{cell}}_{\mathrm{m}},{\mathrm{p}}}} \end{array}} \right] = \left[ {\begin{array}{*{20}{c}} {N_{{\mathrm{cell}}_1,1}} & 0 & \ldots & 0 & 1 \\ {N_{{\mathrm{cell}}_1,2}} & 0 & \ldots & 0 & 1 \\ {N_{{\mathrm{cell}}_1,3}} & 0 & \ldots & 0 & 1 \\ \ldots & \ldots & \ldots & \ldots & 1 \\ \ldots & \ldots & \ldots & \ldots & 1 \\ 0 & {N_{{\mathrm{cell}}_2,1}} & \ldots & 0 & 1 \\ 0 & {N_{{\mathrm{cell}}_2,2}} & \ldots & 0 & 1 \\ 0 & {N_{{\mathrm{cell}}_2,3}} & \ldots & 0 & 1 \\ \ldots & \ldots & \ldots & \ldots & \ldots \\ 0 & 0 & \ldots & {N_{{\mathrm{cell}}_{\mathrm{m}},{\mathrm{p}}}} & 1 \end{array}} \right] \cdot \left[ {\alpha _{{\mathrm{cell}}_1}\;\alpha _{{\mathrm{cell}}_2}\; \ldots \;\alpha _{{\mathrm{cell}}_{\mathrm{m}}}\;\beta } \right]^{\mathrm{T}}$$

where *I*_cell1,1_ is the measured metabolite intensity in sample 1 of cell line 1, *N*_cell1,1_ is the corresponding number of cells extracted in sample 1 of cell line 1. Cell line specific *α*s and *β* are the unknown parameters to be fitted. We selected the metabolites exhibiting a significant alteration in at least one cell line using a one-way ANOVA test, including a step correcting for multiple hypothesis testing^66–68^ (Supplementary Figure 15).

### Data processing and computational analysis for dynamic drug-induced metabolome changes

The full matrix of dynamic metabolic profiles after DCA and oxamate treatments is provided in Supplementary Data 2. In order to deduce a specific metabolic fingerprint induced by an external perturbing agent, we first selected the most significant metabolic changes conserved across the cell lines, performed pathway enrichment analysis on the resulting list of metabolites, and lastly analyzed response variability across all cell lines.

Separately for each perturbation (i.e. oxamate and DCA), we extracted the most significant and prominent metabolic changes that are conserved across the different cell lines. To this end, for each individual metabolite time course we calculated the median of maximum absolute fold changes and the product of lowest *p*-values across cell lines. As a result, each metabolite is associated with a unique median fold-change and *p*-value, summarizing the effect of the perturbation on all cell lines.

Metabolites with an absolute log_2_ fold-change ≥ 1 and a combined *p*-value ≤ 1e−10 were then tested against KEGG metabolic pathways. Pathways with an overrepresented number of altered metabolites were selected based on a hypergeometric statistical test and *p*-value correction for multiple tests^66,67^.

Metabolites that exhibit cell line-specific responses to a given perturbation were selected on the basis of the response variability exhibited across the different cell lines. The standard deviation for each metabolite was calculated from the aforementioned maximum fold changes in each cell-line time course, and metabolites with a standard deviation ≥ 1.5 are retained and subjected to pathway enrichment analysis (Supplementary Figure 15).

### Minimal kinetic model

Here we describe cell proliferation as a function of CoA biosynthesis, assuming that DCA activates CoA biosynthesis and CoA inhibits growth. Reactions for the production (*v*_CoA_) and consumption (*v*_biomass_) of CoA follow a Michaelis–Menten type of kinetics (see Eqs. (4) and (5) in the main text).

The two key assumptions are that CoA is not limiting for biomass production (i.e. [CoA] ≫ *K*_m_), and that cells are ultrasensitive to high levels of CoA, which in turn inhibit biomass production (i.e. *K*_i_ ≫ *K*_m_). To simulate the effect of a reduced CoA biosynthesis as a function of hopantenate (HoPan) we divided *v*_CoA,max_ by HoPan concentrations. In the simulations reported in Fig. 5, we used the following parameters: *v*_biomass,max _= 1, *K*_CoA_ = 0.01, *K*_i_ = 1, *v*_CoA,max_ = 0.45, [CoA]_0_ = 10, [DCA] = 0.01, and [HoPan] = 5. The system of differential equations was solved using the SymBiology toolbox in Matlab 2015. It is worth noting that qualitatively the model would behave similarly if we assumed that the growth inhibitory compound is not the total pool of CoA, but an intermediary toxic compound of CoA metabolism that rapidly equilibrates with the CoA pool.

### Quantification of pantothenate using LC–MS/MS

SKOV3 cells were seeded in RPMI-1640 medium in six-well plates, and supplemented with 11.3 mM DCA in RPMI-1640, or an equal volume of medium without DCA. After 24 h, cells were washed once with 75 mM ammonium carbonate (pH 7.4, 37 °C) and extracted with 500 µL extraction solvent (40% acetonitrile, 40% methanol, 20% water, 25 µM phenyl hydrazine), pre-cooled to −20 °C. The six-well plates were sealed, incubated at −20 °C for one hour, and then stored at −80 °C until further processing. Prior to LC–MS/MS measurements, the plates were thawed, and cells were detached from the bottom of each well using a cell culture scraper. The extract was transferred to separate sample tubes and centrifuged for 5 min at 13,000 rpm to separate cell debris. The supernatants were then transferred to fresh sample tubes, supplemented with equal volumes of fully ^13^C-labeled extract of *Escherichia coli* (prepared in-house), and subsequently dried by vacuum centrifugation. Standard solutions containing different concentrations of pantothenate (d-pantothenic acid calcium salt, Fluka 21210) were prepared similarly, i.e. supplemented with ^13^C-labeled cell extract and dried by vacuum centrifugation. Immediately prior to analysis, all samples were reconstituted in water (10× concentrated) and kept on ice until analysis. Chromatographic separation and MS/MS detection on a triple quadrupole mass spectrometer was performed as described in detail in Buescher et al. ^69^, using an injection volume of 10 µL.

### Data availability

All data generated or analyzed during this study are included in this published article as supplementary data.

## Electronic supplementary material

Supplementary Information

Description of additional Supplementary Infomation

Supplementary Data 1

Supplementary Data 2
