# Supplementary material for: A framework for large-scale metabolome drug profiling links coenzyme A metabolism to the toxicity of anti-cancer drug dichloroacetate
Source: Commun Biol. 2018 Aug 3;1:101. doi: 10.1038/s42003-018-0111-x (PMC6123704; doi:10.1038/s42003-018-0111-x)
Supplement: Supplementary file 1 — Supplementary Information [file 42003_2018_111_MOESM1_ESM.pdf]

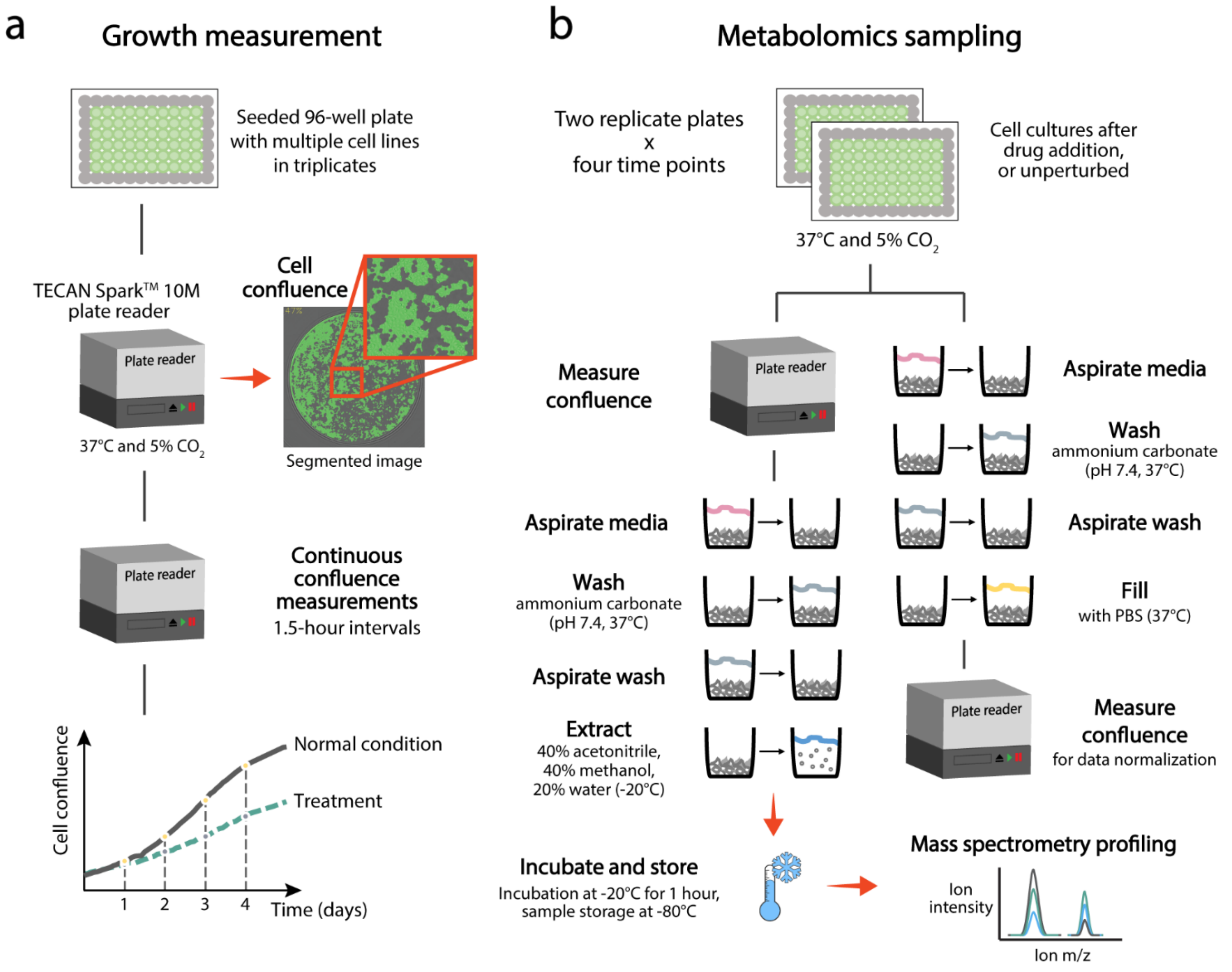

Supplementary Figure 1. Schematic representation of the experimental workflow for dynamic metabolome profiling, adapted from[1]. Experiments using our framework can easily accommodate up to five cell lines screened in parallel in four different conditions, or one cell line screened in up to 20 different conditions, in three replicates, respectively. As such, the setup is also ideally suited to append a panel of reference treatments to cross-reference metabolic responses elicited by uncharacterized test compounds with metabolic phenotypes of interest. **(a)** A TECAN Spark 10M plate reader is used to monitor cell growth by automatically acquiring bright-field images of a 96-well microtiter plate. Confluence was estimated on-line by segmenting the percentage area of the well covered by cells (i.e. green area), using a manufacturer-proprietary algorithm. (**b)** Schematic workflow for metabolome sampling procedure. Two replicate plates are processed at each sampling time-point: one to collect cell extracts (left branch) and a second one to measure the extracted cell number, necessary for subsequent normalization of metabolite intensities (right branch). Metabolome profiles are generated using a non-targeted mass spectrometry platform, while cell numbers are estimated from automated microscopy imaging.

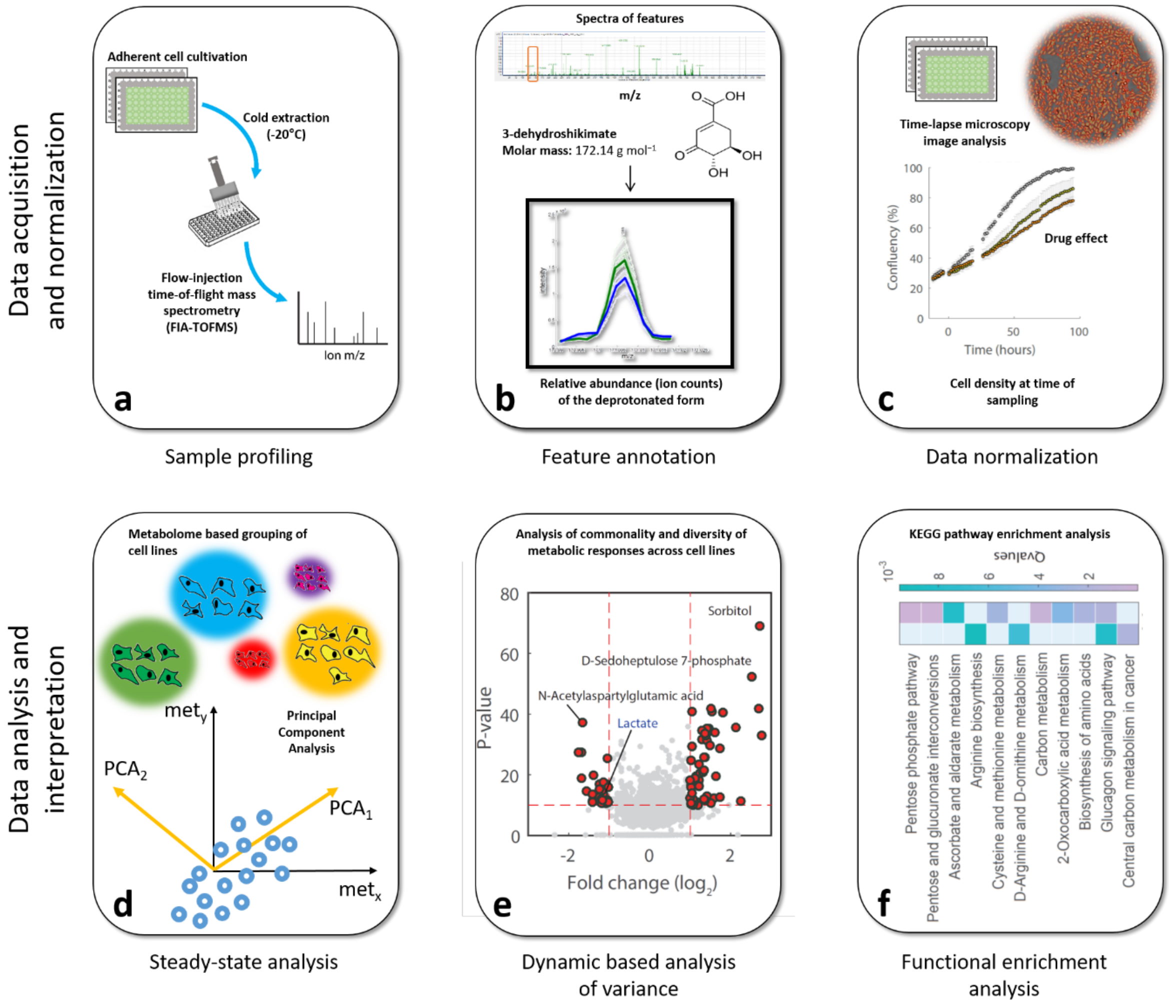

Supplementary Figure 2. Schematic description of MS data normalization and analysis. **(a)** To profile dynamic metabolic changes in mammalian cells induced by small-molecule treatments, we adapted an experimental workflow from [1] and used flow-injection analysis coupled to time-of-flight mass spectrometry (TOFMS) as described in [2]. This method omits chromatographic separation and allows the automated injection of cell extract samples into a constant solvent flow and direct delivery to an Agilent 6550 QTOFMS instrument. **(b)** We annotated detected ions to known metabolites listed in Recon2 and HMDB (for the full list of annotated metabolites see Supplementary Data 1) based on their accurate mass and by assuming that deprotonation is the most frequent and reliable form of ionization in negative mode. **(c)** To normalize raw MS spectra a linear model was used to decouple the variance in the data caused by differences in extracted cell numbers (quantified by means of time lapse microscopy), intrinsic noise of the instrument and drug treatment effects. It is worth noting, that because we estimate relative changes of metabolites within the same cell lines after drug exposure, we can directly adopt confluence measurements to correct for differences in the amount of extracted cells between time-points. Compared to alternative measurements of cell numbers[3], cell volume[4] and cell protein content[5], time-lapse microscopy offers accurate measurements of confluence and bypasses the need for additional experimental steps (e.g. staining, trypsinization). **(d)** Data analysis and interpretation consists of three main steps. To extract differences in the baseline metabolite levels across the different cell lines, we first group cell lines based on the similarity in their metabolome profiles using Spearman pairwise correlation and an affinity propagation algorithm[6]. In parallel, principal component analysis is used to identify the major patterns in the data. Metabolites associated to each principal component (PC) are individuated by calculating the Spearman correlation between each metabolite and PC. We retained only those ions with a Spearman correlation larger or equal to 0.7 to at least one PC. **(e)** Secondly, we investigate commonality and diversity in the metabolic response across cell lines to the same treatments. In the former case, for each individual metabolite time course we calculated the median of the maximum absolute fold changes and the product of lowest p-values across cell lines. On the other hand, Metabolites that exhibit cell line-specific responses to a given perturbation are selected on the basis of their

response variability across different cell lines. The standard deviation for each metabolite is calculated from the aforementioned maximum fold changes, and metabolites with a standard deviation ≥ 1.5 are retained. **(f)** Metabolites associated to the aforementioned groups are tested against KEGG metabolic pathways to search for overrepresented metabolic processes. Significance is estimated by classical hypergeometric tests and correction for multiple test was applied[7].

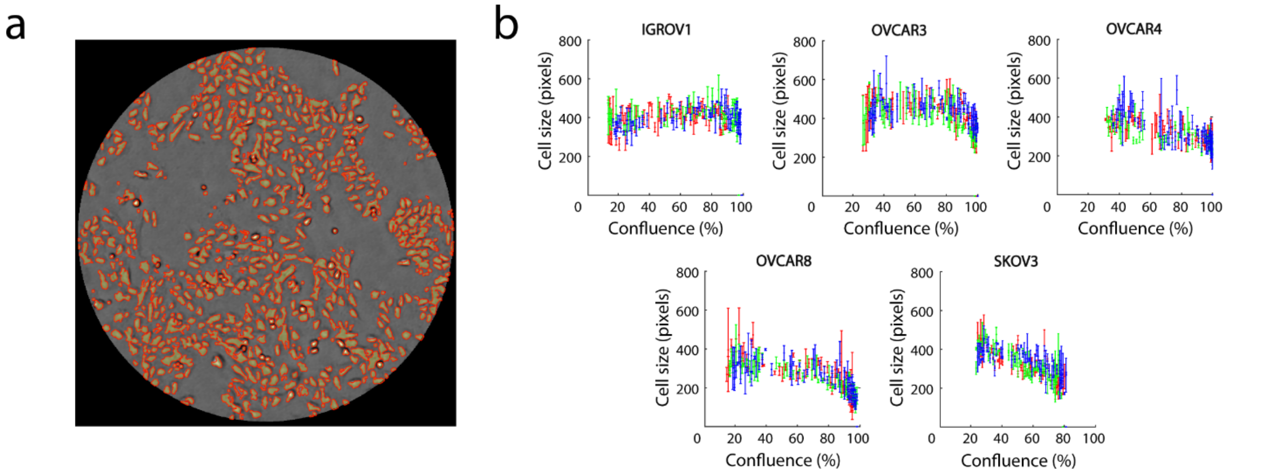

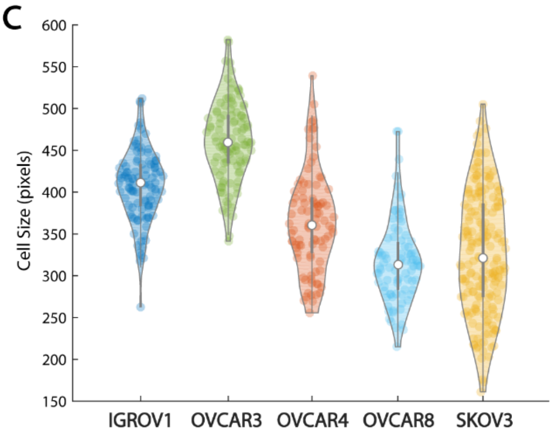

d

Cell line	Area (A, µm²)	A std (µm²)	Radius (r, µm)	r std (µm)
IGROV1	501.23	52.47	8.32	1.88
OVCAR3	590.66	69.52	7.03	1.66
OVCAR4	505.47	65.36	7.73	1.83
OVCAR8	439.15	65.74	7.11	1.35
SKOV3	509.81	59.70	8.60	1.73

Supplementary Figure 3. Determination of average adherent cell sizes. (a) Illustrative example from segmented cells using the image analysis tool described in [1] applied to bright-field microscopy images of adherent ovarian cancer cells in a 96-well plate. Segmented cells are highlighted in green, and cell borders in red. **(b)** Dependence of cell size on cell confluence in three biological replicates of the five ovarian cancer cell lines (untreated condition). Cell size estimates in each time-lapse frame are plotted against cell confluence. The procedure used to analyze and segment bright field images from TECAN Spark 10M automated plate reader is described in [8]. Here, the average cell size during unperturbed growth remained largely constant in time, up to a confluence of approximately 80%. Above 80% confluence, cells become difficult to segment and we observed a slight cell shrinkage. As a consequence, reliably estimating the extracted cell number above 80% cell confluence is difficult, and we excluded all metabolome measurements taken above this cell density threshold from data analysis. **(c)** Violin plot of the estimated cell sizes in pixels (one pixel corresponds to 1.3 μm^2) for each acquired image with a cell confluence between 20 and 50%. **(d)** The first two columns report the average and standard deviation of cell size estimates from data reported in panel **c**. Additionally, the radius of suspended cells after trypsinization measured in [5] is reported.

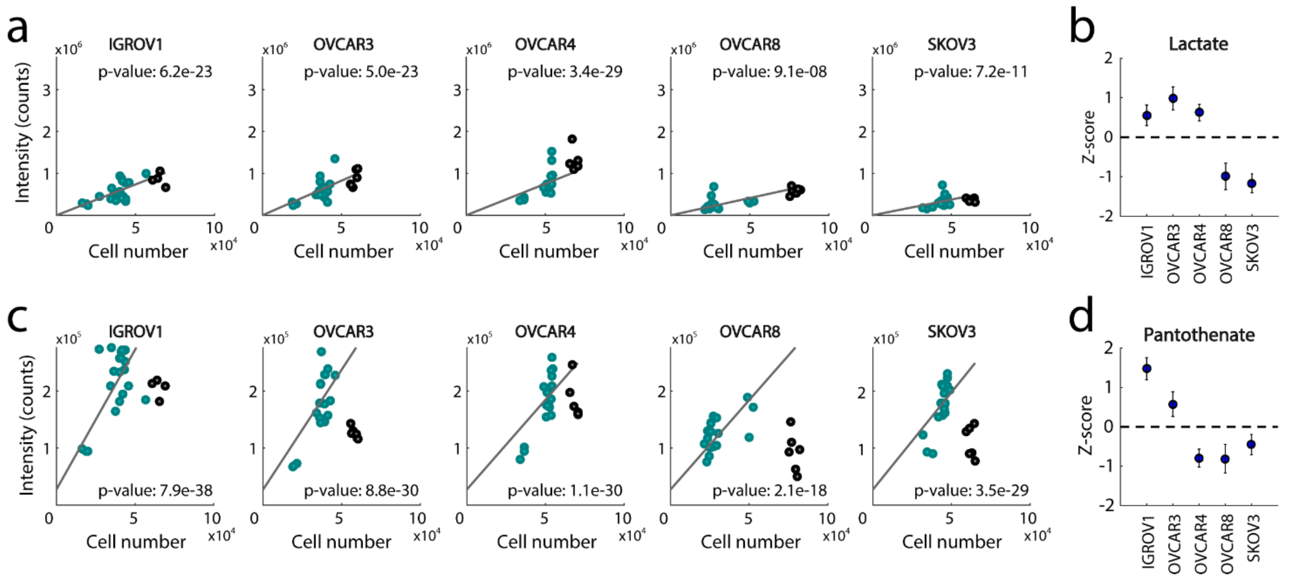

Supplementary Figure 4. Estimation of relative intracellular abundance of metabolites, demonstrated on the example of the metabolites lactate (panels **a-b**), and pantothenate (panels **c-d**). For each ovarian cancer cell line, measured raw intensities of (green dots) are plotted against extracted cell numbers. The bold grey line is the result of the multiple regression scheme adopted to infer the slope (α) of the dependency between measured ion intensities and cell numbers in each cell line. Differences in α-values estimated across cell lines reflect differences in the relative intracellular abundance of lactate per cell. P-values indicate the significance of the regressed parameters. Grey dots represent measured intensities from samples above 80% confluence, which were excluded due to difficulties in reliably estimating extracted cell numbers above 80% confluence. Panels **b** and **d** show the resulting steady-state differences in relative metabolite abundances across five ovarian cancer cell lines. Relative metabolite abundances are given as Z-scores of estimated α-values, i.e. as the deviation from the mean metabolite abundance across cell lines, standardized by the standard deviation of metabolite abundance across cell lines. Error bars reflect the standard error of estimated α-values.

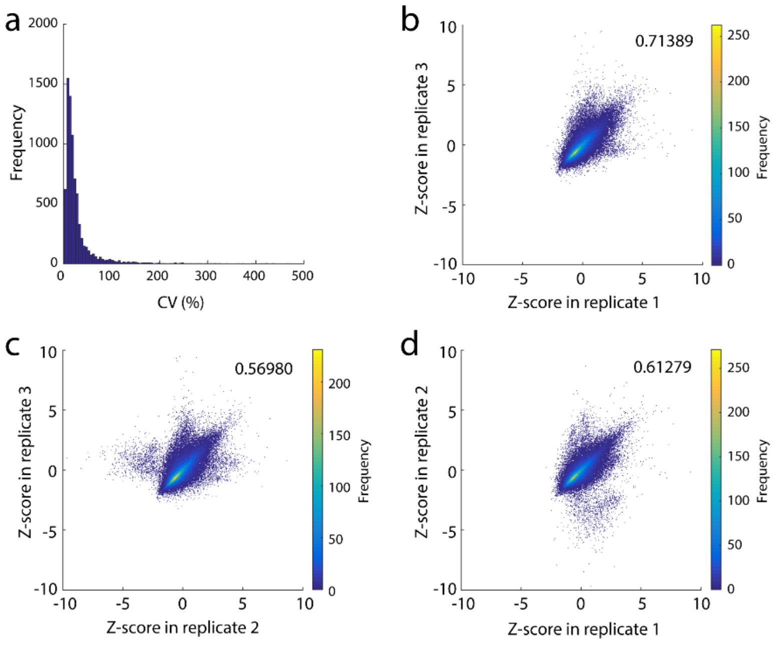

Supplementary Figure 5. Similarity of relative metabolite abundances across three biological replicates. (a) Distribution of calculated coefficients of variation in α-values across three replicates. The error estimated from the linear fit of α-values at steady state (i.e. unperturbed) is divided by the corresponding estimates of α. **(b-d)** Analysis of correlations across biological replicates. For each metabolite, raw MS data across the three biological replicates are rescaled using Z-score normalization. Pearson correlation between pairs of biological replicates was estimated by comparing Z-score intensities across all cell lines and conditions (reported on the upper-right corner of each subplot). The robustness and repeatability of metabolome profiles obtained with our experimental platform is illustrated by bivariate histograms (the matlab function histogram2 was used). In each plot, Z-score values from two biological replicates are plotted against each other. The 2D-histograms reveal the underlying shape and density of the distribution and give a visual perspective on the correlation between replicates. In in each panel, we reported ~100.000 points corresponding to the normalized Z-score of all annotated ions across all conditions and time points. There is only a minor fraction (< 5%) of metabolites that exhibit a difference in Z-score between two replicates larger than 2.

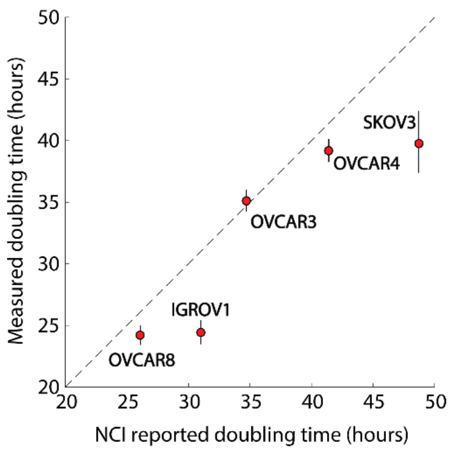

Supplementary Figure 6. Cell line doubling times. For each cell line in normal untreated condition, the doubling time was estimated from the exponential fit of confluence measurements between 20 and 80% (Figure 1 in the main text). The plot shows a comparison of our growth rate estimates to previously experimentally measured ones[9]. Overall, the determined growth rates of the five ovarian cancer cell lines vary from 20 to approximately 40 hours doubling time, and are in good agreement with previous NCI growth-rate measurements. It is worth noting that we used dialyzed FBS as a medium supplement. The absence of the low-molecular weight fraction of the serum might explain the observed slight differences in growth rates.

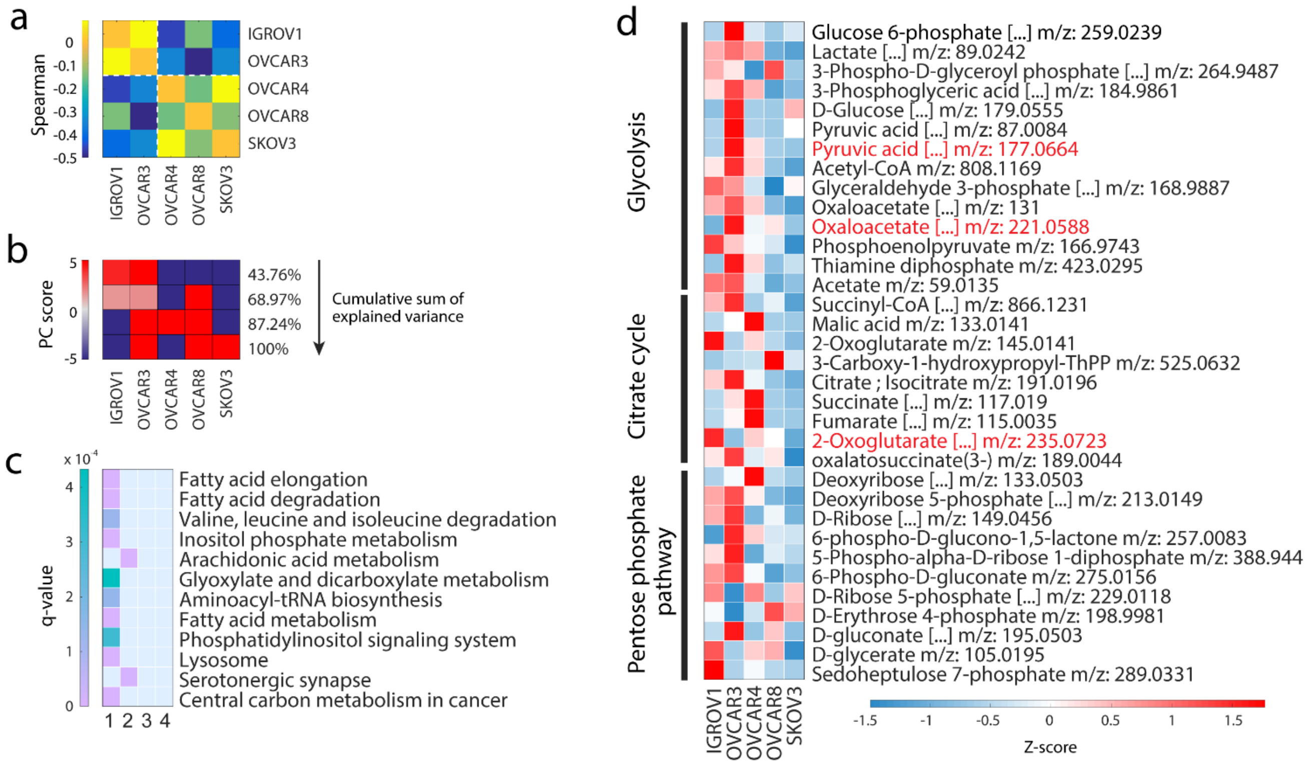

Supplementary Figure 7. Analysis of steady-state metabolic profiles in ovarian cancer cells. (a) Spearman pairwise metabolome similarity across the five cell lines. Spearman correlation was calculated between Z-score normalized metabolome profiles of cell line pairs. To avoid any a priori definition of the number of clusters, we adopted an affinity propagation algorithm[6] . The affinity propagation algorithm detected two major groups of cell lines based on metabolome similarity, separated by the dashed white lines. **(b)** Principal component analysis results. In order to identify major trends in the full metabolome data set, we performed principal component analysis (pca function in Matlab) and subsequently determined four groups of metabolites, by correlating metabolite levels in the five cell lines to each individual principal component (Spearman correlation ≥ 0.7) (Table S1). The first principal component explains 43% of the variance in the data. **(c)** Pathway enrichment analysis. Each row corresponds to a metabolic pathway, while each column represents metabolites associated to one of the four principal components. We tested the four groups of metabolites identified in step **(b)** for an overrepresentation of metabolites in KEGG metabolic pathways, using a hypergeometric test and p-value correction for multiple tests (pathway enrichment analysis)[7,10] Only pathways with q-value ≤ 0.001 are considered. The first two components exhibit statistically significant enrichment for metabolites in specific metabolic pathways, while the remaining PCs consist of more heterogeneous metabolites, and no statistical enrichment for metabolic pathways was identified. **(d)** Z-scores of α-values associated to metabolites in central carbon metabolism. Metabolites marked in red are α-keto acids that were detected as phenyl hydrazine derivatives.

The analysis of relative metabolite abundances (α-values) across the five ovarian cancer cell lines revealed heterogeneous metabolome patterns. We detected an overall tendency to form two main groups, differentiating OVCAR3 and IGROV1 from OVCAR4, OVCAR8 and SKOV3 (panel **a**). Principal component analysis revealed that a large portion of metabolites exhibited a larger pool size in OVCAR3 and IGROV1 with respect to the three remaining ovarian cancer cell lines (i.e. first principal component in panel **b**). Furthermore, we found that metabolites with higher levels in OVCAR3 and IGROV1 were significantly overrepresented (q-value ≤ 0.001) in fatty acid and central carbon metabolism (panel **c-d**). Recent studies[11,12] have unraveled that more invasive ovarian cancer cells, like OVARC8 and SKOV3, exhibit a drastically different utilization of nutrients with respect to low-invasive OVCAR3 and IGROV1 cells. In particular, high-invasive ovarian cancer cells depend on glutamine as an essential anaplerotic substrate to drive tricarboxylic acid (TCA) cycle[11,12]. We found that intermediates in glycolysis (e.g. glyceraldehyde 3-phosphate, phosphoenolpyruvate) and TCA cycle (e.g. citrate, 2-oxoglutarate) are more abundant in low-invasive than in high-invasive ovarian cancer cells (panel **d**). This finding is consistent with previous data[12], and possibly reflect the described shift from glycolysis to oxidative phosphorylation in high-invasive cancer cells[12]. Moreover, pathway enrichment analysis revealed major differences in fatty acid

metabolism (panel **c**), in agreement with an up-regulation of monoglycerol lipase and fatty acid metabolism in invasive cancer cells such as SKOV3 and OVCAR8[13]. Overall, our method is capable of recapitulating several metabolic features differentiating invasive from non-invasive ovarian cancer cells, and expands cell line characterization by a multi-parametric phenotypic readout comprising several hundreds of metabolic species.

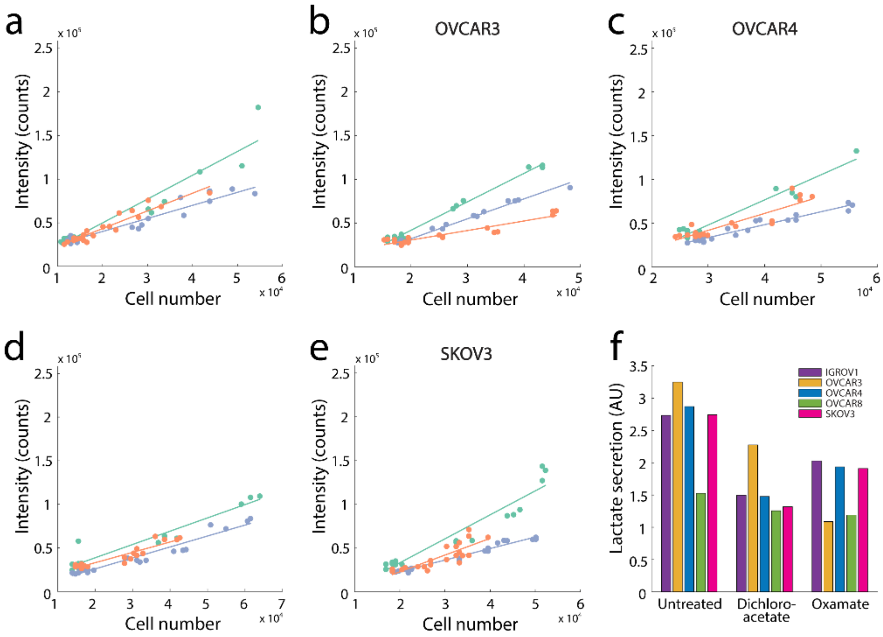

Supplementary Figure 8. Lactate secretion rates. (a-e) Supernatant samples were collected from cell cultures of the five ovarian cancer cell lines grown in RPMI1640 medium (untreated), and after exposure to dichloroacetate and oxamate. Samples were analyzed by FIA-TOFMS. Relative concentrations of lactate in the supernatant were fitted using linear least squares regression analysis. **(f)** The slopes of the fitted lines reflect a relative measure of lactate secretion rates, which are reported in the bar plot.

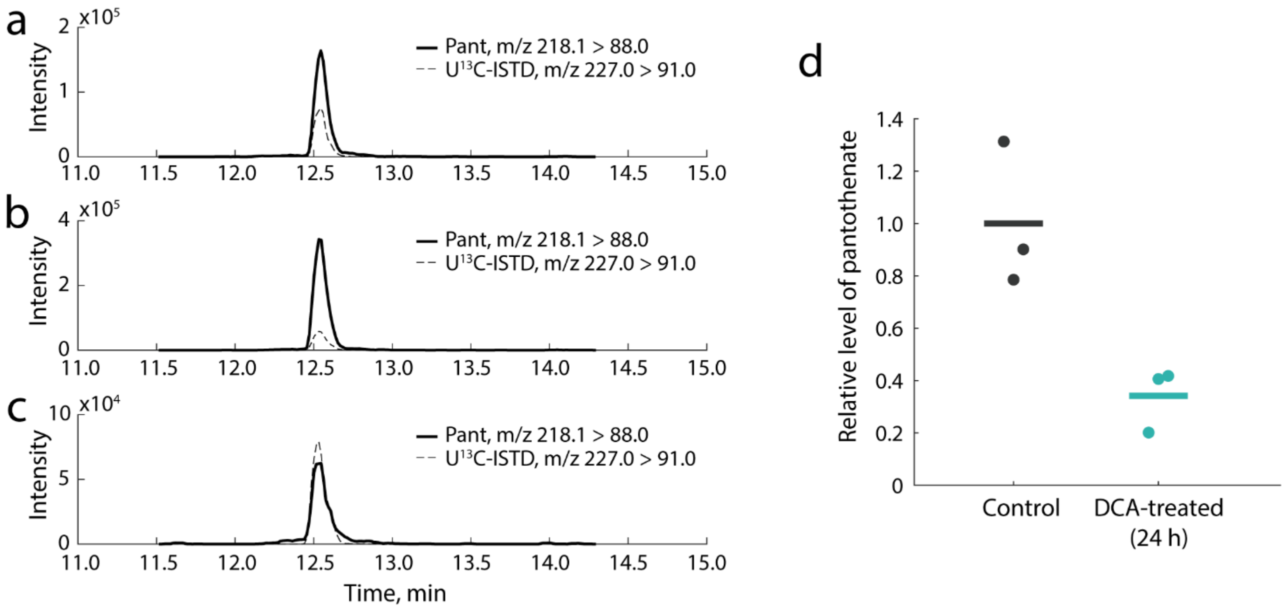

Supplementary Figure 9. Relative quantification of pantothenate in SKOV3 cell extracts using LC-MS/MS. (a-c) Pantothenate (bold line) and the corresponding fully ^{13}C-labeled pantothenate (dashed line, internal standard) were detected at a retention time of 12.53 min in **(a)** a standard solution (0.2 µM pantothenate), **(b)** a cell extract sample of untreated SKOV3 cells, and **(c)** a cell extract sample of SKOV3 cells treated with 25 mM dichloroacetate for 24 hours. **(d)** Comparison between intracellular levels of pantothenate in SKOV3 cells treated with dichloroacetate (DCA) for 24 hours and in untreated SKOV3 cells (Control). We confirmed a more than three-fold reduction in pantothenate, in agreement with the relative changes observed in non-targeted metabolomics data obtained with FIA-TOFMS (Figure 3 in the main text). Pantothenate levels in three biological replicates are given relative to the mean level of the control condition, with the bold representing the mean across replicates.

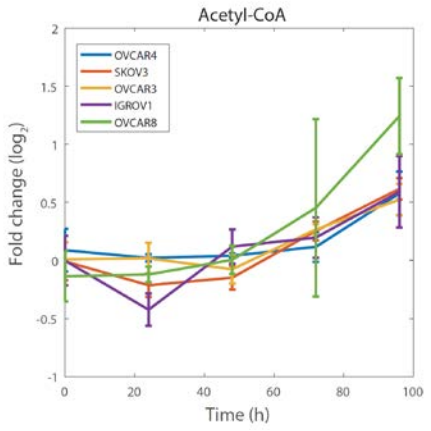

Supplementary Figure 10. Dynamic profiles for acetyl-CoA upon dichloroacetate treatment. Data are mean and SD of 3 biological replicates.

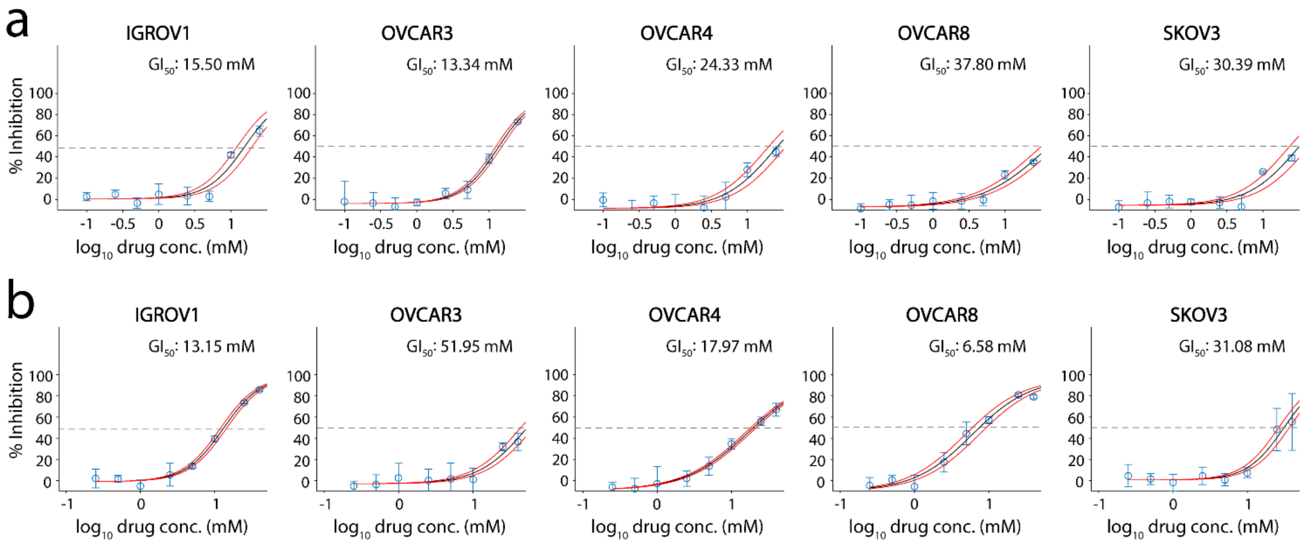

Supplementary Figure 11. GI50 determination for dichloroacetate (panel a) and oxamate (panel b) in five ovarian cancer cell lines. For each cell line, the growth rates at different drug concentrations relative to growth in the untreated condition were estimated. A sigmoidal curve was fitted to the data and the GI_{50} is estimated as the concentration that reduces the growth rate by 50%. While the metabolic response to dichloroacetate treatment (panel **a**) is qualitatively consistent across the five ovarian cancer cell lines, we observed differences in the sensitivity to dichloroacetate. According to our hypothesis on the mode of action of dichloroacetate (see main text Figure 3d), differences in dichloroacetate sensitivity could be determined by the basal state of CoA metabolism. We observed the highest pantothenate levels and the lowest expression of intermediate enzymes in the pathway[14] (Supplementary Figure 13) in OVCAR3 and IGROV1, the two cell lines with the lowest GI50 values (see also main text Figure 3). This may reflect a strong repression of PANK2 and CoA biosynthesis and could indicate that these cells maintain a higher basal level of CoA in the mitochondria. Such a metabolic phenotype appears to predispose cells to dichloroacetate senitivity (Figure 3a-b), potentially by aggravating the cells' efforts to restore the CoA balance. While it has been proposed that the PDH reaction operates far from equilibrium under normal physiological conditions[15], high proliferation rates and dichloroacetate can shift the equilibrium by increasing the total amount of unphosphorylated PDH[14]. Hence, higher baseline levels of CoA in the mitochondria can potentially cause enhanced sensitivity to the drug in cell lines with higher basal CoA (Figure 3a-b).

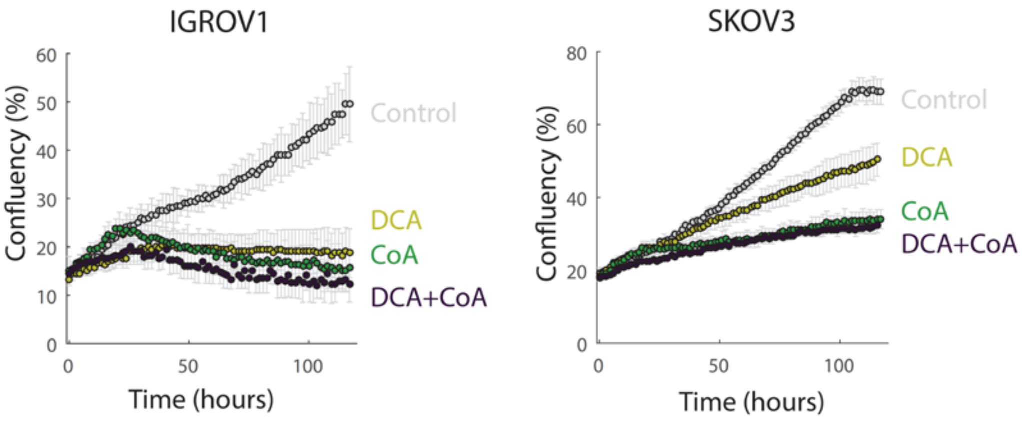

Supplementary Figure 12. Phenotypic characterization of IGROV1 and SKOV3 cell lines. Growth of IGROV1 and SKOV3 cell lines was monitored for ~5 days by time-lapse microscopy using a TECAN Spark 10M plate reader. Cells were grown for 24 hours in normal RPMI1640 medium before addition of perturbing agents and continuous confluence monitoring. Four different conditions were tested: normal RPMI1640 medium (Control), addition of 500 µM CoA with and without 11 mM (IGROV1) or 25 mM (SKOV3) dichloroacetate (CoA/CoA+DCA). Cell confluence is reported as mean ± SD across three replicates.

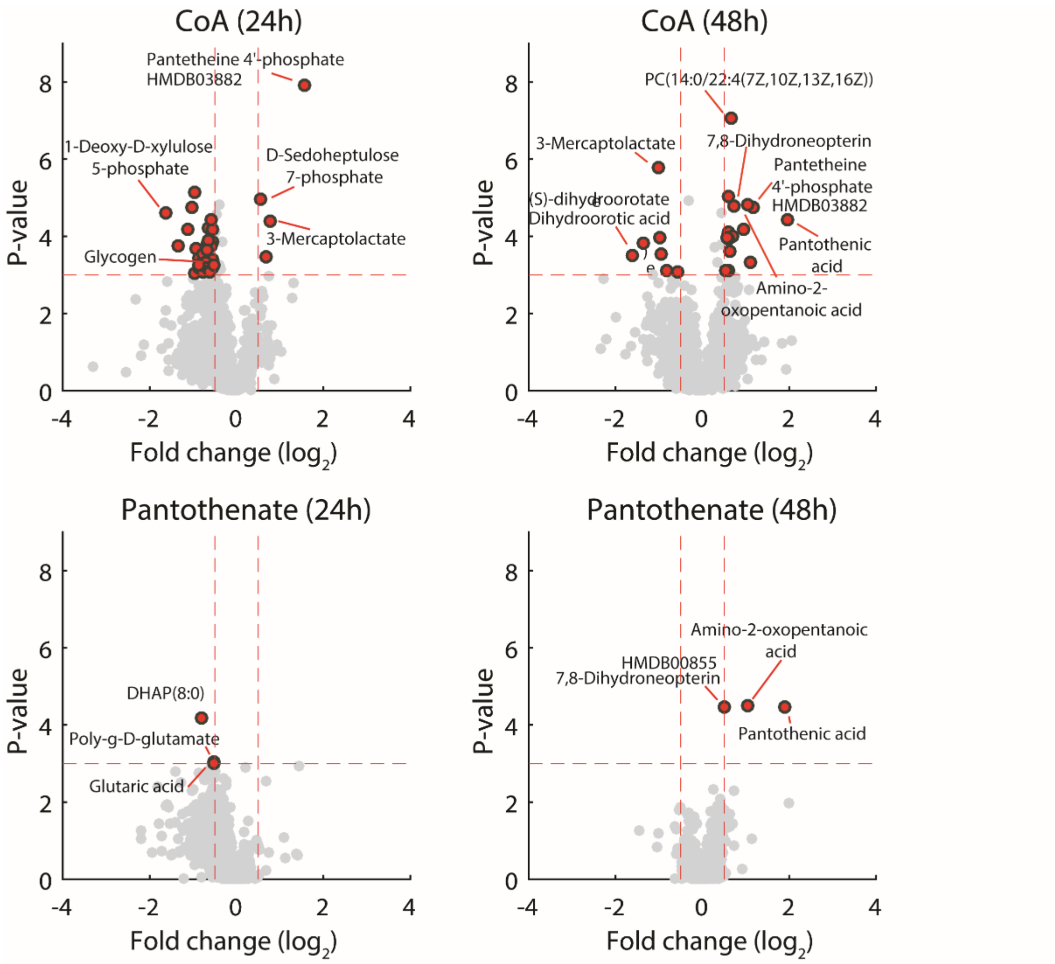

Supplementary Figure 13. Metabolic changes in IGROV1 cells upon addition of 2.1 µM pantothenate, or 200 µM CoA. Volcano plots of metabolic changes induced 24 and 48 hours after addition of 200 µM CoA and 2.1 µM pantothenate. As hypothesized, adding pantothenate induces negligible perturbation in intracellular metabolite levels, possibly because cells can regulate CoA biosynthesis at the level of pantothenate kinase (PANK) to maintain CoA homeostasis in the cell. In contrast, addition of 200 µM CoA causes pleiotropic metabolic changes as cells are no longer able to control for CoA biosynthesis. Consistent with our expectations we found a drastic intracellular accumulation of pantetheine 4'-phosphate and a subsequent accumulation of pantothenate, which is consistent with increased levels of CoA inhibiting PANK enzymes.

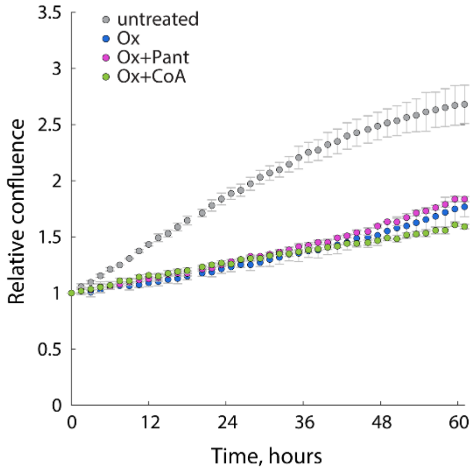

Supplementary Figure 14. Growth of IGROV1 cells treated with oxamate, in presence or absence of pantothenate and CoA, respectively. Cells grown in RPMI1640 medium (untreated, grey curve) were treated with the GI50 concentration of oxamate (12.5 mM, blue curve), and additionally supplemented with 2.1 µM pantothenate (pink curve) or 200 µM CoA (green curve). Growth was monitored continuously by time-lapse microscopy using a TECAN Spark 10M plate reader. Cell confluence is reported as mean ± SD across three replicates, relative to the initial cell confluence.

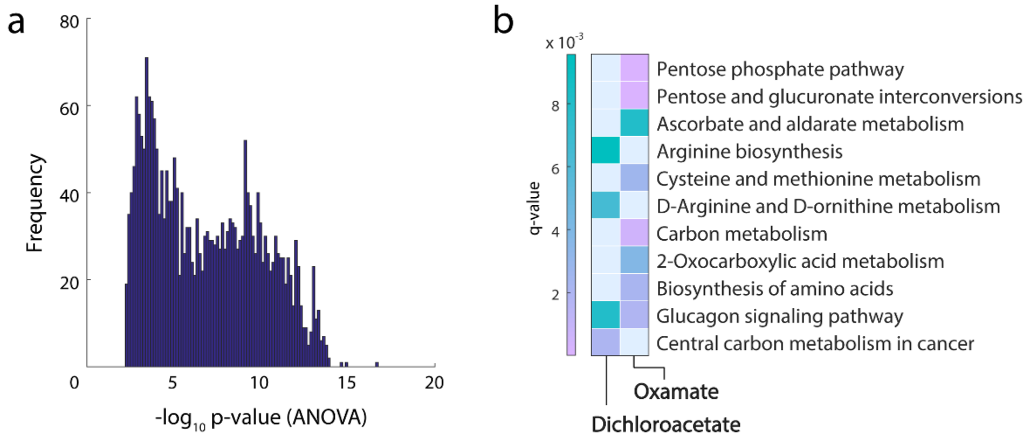

Supplementary Figure 15. Metabolomics data analysis. (a) Distribution of p-values from one-way ANOVA test. All annotated metabolites with a significant estimate of α (p-value <= 0.001, and α > 0 in at least one of the five ovarian cancer cell lines) were tested for significant variation across cell lines, using an ANOVA test. The resulting p-values and their distribution are reported in the histogram. **(b)** KEGG pathway enrichment among metabolites that exhibit high response variability across cell lines. Metabolites exhibiting cell line-specific responses to a given perturbation are selected as follows: the standard deviation for each metabolite is calculated from maximum fold changes over the time course and across cell lines. Metabolites with a standard deviation ≥ 1.5 were retained and subjected to pathway enrichment analysis. Only pathways with q-values ≤ 0.001 are shown. Several metabolites exhibited cell line-specific responses to oxamate, these predominantly include intermediates of the pentose phosphate pathway and several keto acids, a family of metabolites with antioxidant properties that are able to scavenge reactive oxygen species[16,17]. This group of metabolites can hint at intrinsic tolerance mechanisms and possibly explain the differences in GI_{50} among cell lines.

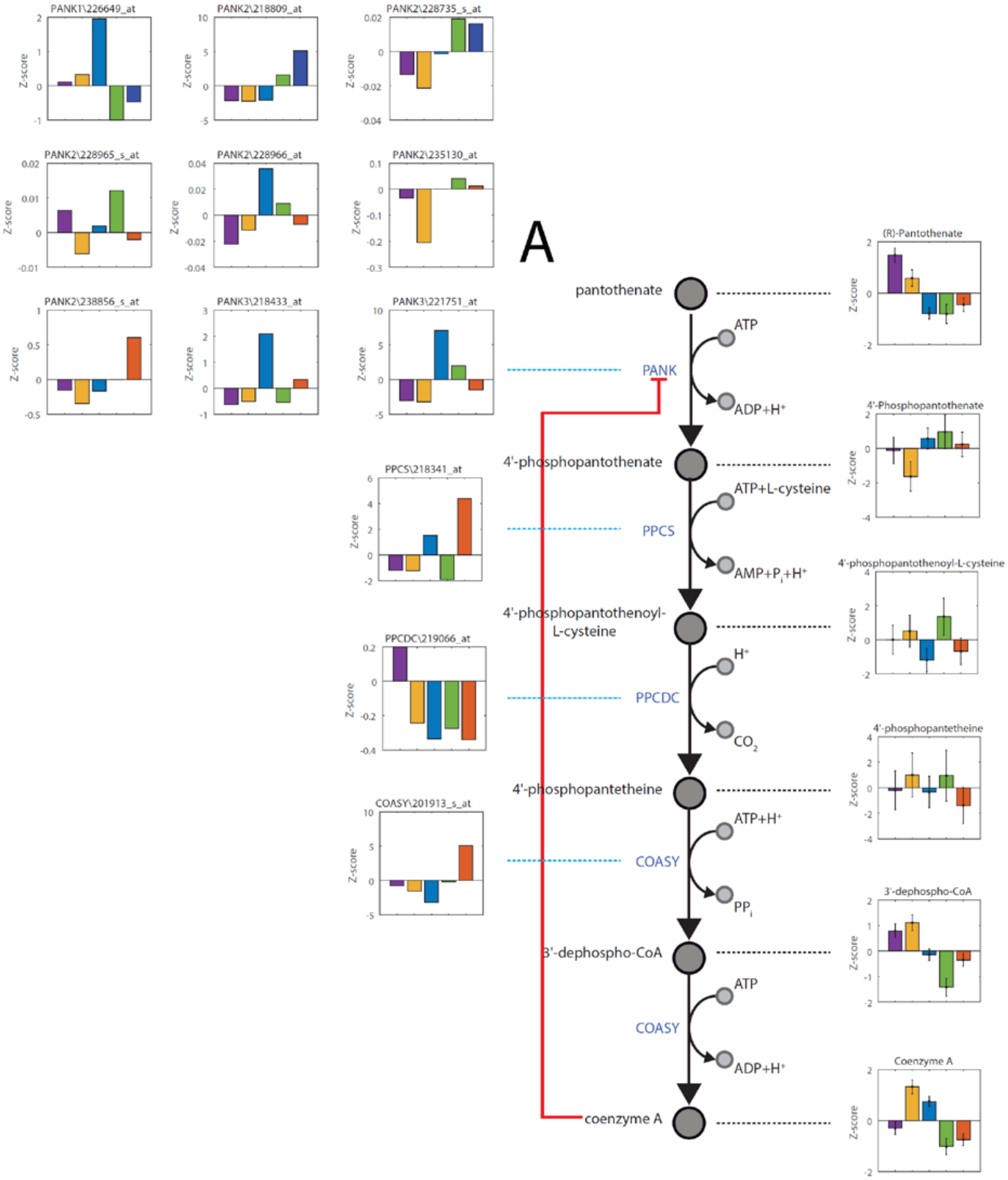

Supplementary Figure 16. Schematic representation of the CoA biosynthetic pathway. Pantothenate kinases (PANK) catalyze the rate-limiting step in CoA biosynthesis[18]. Relative steady-state abundances of pathway intermediates in untreated condition are reported on the right hand side of the pathway scheme. For each enzyme, z-score normalized mRNA levels from[19] are reported on the left-hand side. Each bar represents a different ovarian cancer cell line. The differences in baseline levels of metabolites and transcripts within the CoA biosynthetic pathways are indicative of potential differences in CoA biosynthesis. Cells seem to tightly allosterically and transcriptionally regulate CoA biosynthesis and its compartmentalization. This tight regulation might be linked to the toxic role of imbalanced CoA. IGROV1 and OVCAR3 which are more sensitive to high levels of DCA (Figure 3), seem to have a reduced CoA biosynthesis. We deduce this from the observed high level of pantothenate and lower level of biosynthetic enzymes, potentially reflecting the reduced CoA biosynthetic flux. Under such conditions, modulation of intracellular CoA levels either by external CoA, or DCA can cause an even more dramatic change from baseline and consequently have more severe effects.

Supplementary references

1. Ortmayr, K., Dubuis, S. & Zampieri, M. Charting the cross-functional map between transcription factors and cancer metabolism. *bioRxiv* 250399 (2018). doi:10.1101/250399
2. Fuhrer, T., Heer, D., Begemann, B. & Zamboni, N. High-Throughput, Accurate Mass Metabolome Profiling of Cellular Extracts by Flow Injection–Time-of-Flight Mass Spectrometry. *Anal. Chem.* **83,** 7074–7080 (2011).
3. Silva, L. P. *et al.* Measurement of DNA Concentration as a Normalization Strategy for Metabolomic Data from Adherent Cell Lines. *Anal. Chem.* **85,** 9536–9542 (2013).
4. Milo, R. What is the total number of protein molecules per cell volume? A call to rethink some published values. *BioEssays* **35,** 1050–1055 (2013).
5. Dolfi, S. C. *et al.* The metabolic demands of cancer cells are coupled to their size and protein synthesis rates. *Cancer Metab.* **1,** 20 (2013).
6. Frey, B. J. & Dueck, D. Clustering by Passing Messages Between Data Points. *Science* **315,** 972–976 (2007).
7. Storey, J. D. A direct approach to false discovery rates. *J. R. Stat. Soc. Ser. B Stat. Methodol.* **64,** 479–498 (2002).
8. Ortmayr, K., Dubuis, S. & Zampieri, M. Charting the cross-functional map between transcription factors and cancer metabolism. doi:https://doi.org/10.1101/250399
9. Shoemaker, R. H. The NCI60 human tumour cell line anticancer drug screen. *Nat. Rev. Cancer* **6,** 813–823 (2006).
10. Storey, J. D. & Tibshirani, R. Statistical significance for genomewide studies. *Proc. Natl. Acad. Sci.* **100,** 9440–9445 (2003).
11. Yuan, L. *et al.* Glutamine promotes ovarian cancer cell proliferation through the mTOR/S6 pathway. *Endocr. Relat. Cancer* **22,** 577–591 (2015).
12. Yang, L. *et al.* Metabolic shifts toward glutamine regulate tumor growth, invasion and bioenergetics in ovarian cancer. *Mol. Syst. Biol.* **10,** 728–728 (2014).
13. Nomura, D. K. *et al.* Monoacylglycerol Lipase Regulates a Fatty Acid Network that Promotes Cancer Pathogenesis. *Cell* **140,** 49–61 (2010).
14. Rock, C. O., Calder, R. B., Karim, M. A. & Jackowski, S. Pantothenate Kinase Regulation of the Intracellular Concentration of Coenzyme A. *J. Biol. Chem.* **275,** 1377–1383 (2000).
15. Park, J. O. *et al.* Metabolite concentrations, fluxes and free energies imply efficient enzyme usage. *Nat. Chem. Biol.* **12,** 482 (2016).
16. Bayliak, M. M., Lylyk, M. P., Vytvytska, O. M. & Lushchak, V. I. Assessment of antioxidant properties of alpha-keto acids in vitro and in vivo. *Eur. Food Res. Technol.* **242,** 179–188 (2016).
17. Kładna, A., Marchlewicz, M., Piechowska, T., Kruk, I. & Aboul-Enein, H. Y. Reactivity of pyruvic acid and its derivatives towards reactive oxygen species. *Luminescence* **30,** 1153–1158 (2015).
18. Jackowski, S. & Rock, C. O. Regulation of coenzyme A biosynthesis. *J. Bacteriol.* **148,** 926–932 (1981).
19. Pfister, T. D. *et al.* Topoisomerase I levels in the NCI-60 cancer cell line panel determined by validated ELISA and microarray analysis and correlation with indenoisoquinoline sensitivity. *Mol. Cancer Ther.* **8,** 1878–1884 (2009).
