# Supplementary material for: A framework for large-scale metabolome drug profiling links coenzyme A metabolism to the toxicity of anti-cancer drug dichloroacetate
Source: Commun Biol. 2018 Aug 3;1:101. doi: 10.1038/s42003-018-0111-x (PMC6123704; doi:10.1038/s42003-018-0111-x)
Supplement: Supplementary file 2 — Description of additional Supplementary Infomation [file 42003_2018_111_MOESM2_ESM.docx]

**Supplementary Data Legends**

**Supplementary Data 1. Steady-state metabolome profiling of the five ovarian cancer cell lines IGROV1, OVCAR3, OVCAR4, OVCAR8 and SKOV3.**

Table “A”: contains the fitted α values for each cell lines

Table “A std”: contains the error in the estimate of α values for each cell lines (i.e. standard deviation among the three biological replicates)

Table “Ap”: contains the significance of the estimate of α values for each cell lines (pvalues)

Table “Az”: Z-score nomalization of A

Table “Az std”: error on the Z-score

**Supplementary Data 2. Dynamic metabolic profiles of drug responses in IGROV1, OVCAR3, OVCAR4, OVCAR8 and SKOV3 cells treated with the anti-cancer drugs dichloroacetate (D) and oxamate (O), respectively.**

Table “D FC”: contains time-dependent metabolite fold-change profiles for each cell line upon dichloroacetate treatment

Table “D FC std”: contains the standard deviations of time-dependent metabolite fold-change profiles for each cell line upon dichloroacetate treatment

Table “D FC pvalue”: contains the p-values (t-test) of time-dependent metabolite fold-changes upon dichloroacetate treatment

Table “O FC”: contains time-dependent metabolite fold-change profiles for each cell line upon oxamate treatment

Table “O FC std”: contains the standard deviations of time-dependent metabolite fold-change profiles for each cell line upon oxamate treatment

Table “O FC pvalue”: contains the p-values (t-test) of time-dependent metabolite fold-changes upon oxamate treatment

Table “Variability analysis”: contains the standard deviation of cell line maximum fold-changes across the five cell lines
